# Environmental and phytohormone modulation of organ‐specific specialized metabolite profiles in the dryland tree *Erythrina velutina*

**DOI:** 10.1111/plb.70104

**Published:** 2025-09-08

**Authors:** D. S. Chacon, B. Bonilauri, C. T. da Costa, J. Vilasboa, M. Koetz, L. Pinto, J. A. S. Zuanazzi, R. B. Giordani, A. G. Fett‐Neto

**Affiliations:** ^1^ Department of Pharmacy Federal University of Rio Grande do Norte (UFRN) Natal Brazil; ^2^ Department of Medicine, Stanford Cardiovascular Institute Stanford University School of Medicine Stanford California USA; ^3^ Department of Botany and Center for Biotechnology, Plant Physiology Laboratory Federal University of Rio Grande do Sul Porto Alegre Brazil; ^4^ Department of Raw Material Production, Faculty of Pharmacy, Laboratory of Pharmacognosy Federal University of Rio Grande do Sul Porto Alegre Brazil; ^5^ Department of Analytical Chemistry (DQA) State University of Rio de Janeiro (UERJ) Rio de Janeiro Brazil

**Keywords:** Caatinga, elicitation, fingerprint, HPLC‐DAD, specialized metabolites

## Abstract

- *Erythrina velutina* is a tree that thrives in the shallow rocky soils of the dry and hot Caatinga, a unique Brazilian biome. It is rich in specialized metabolites with medicinal properties. Indeed, alkaloids and flavonoids are phytochemical markers of the genus. Our previous studies identified key biochemical and molecular targets in biosynthesis of these metabolites in *E. velutina*, including phytohormone signalling pathways and responses to environmental stressors. However, the role of these signalling molecules and external factors in modulating the tree natural product (NP) profiles remains unexplored.
- In this study, seedlings of *E. velutina* were subjected to environmental stress (heat, ultraviolet radiation, drought, salinity, mechanical damage) and phytohormone exposure (methyl jasmonate, salicylic acid, nitric oxide, abscisic acid). Leaves and roots were collected after 2 and 4 days of treatment for HPLC‐DAD and chemometric analyses.
- The most prominent factors that increased accumulation of major metabolites were nitric oxide, drought, heat, ultraviolet radiation, and methyl jasmonate. The analyses revealed both organ‐ and temporal‐specific metabolite profiles, as well as some typically shared features. Both phytoanticipin and phytoalexin‐like metabolite profiles were recorded, with prevalence of the former.
- The results shed light on how the above factors affect metabolic tuning in *E. velutina*. Moreover, the generated datasets will be useful in selecting individual compounds for detailed functional investigation, as well as for directing chemical profiles towards known and novel metabolites of interest in this species.

## Graphical abstract

*Erythrina velutina* organ‐specific specialized metabolism is modulated by environmental stress and phytohormones to mitigate effects of the harsh semi‐arid habitat.

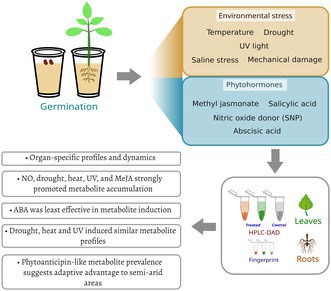

## INTRODUCTION

Plants are rich in specialized metabolites essential for the conquest of diverse environments. The sessile nature of plants drove the evolution of complex biochemistry, affording a remarkable capacity to respond and adapt to environmental challenges. Specialized metabolites provide defence against biotic natural enemies (herbivores and pathogens), attract helpers (tritrophic interactions), and confer allelopathic activity. These metabolites also mediate plant interactions with pollinators, dispersers, and symbionts. Specialized metabolites also provide protection against abiotic stresses by acting as antioxidants, ultraviolet radiation shields, membrane stabilizers, and heat dissipating agents, among other activities (Junkes *et al*. 2019).

Metabolite fingerprints in plant defence are classified according to accumulation profiles (Hartmann 2007) and can be constitutively accumulated, characterizing the phytoanticipin profile. A second profile type, closely related to that of phytoanticipins, involves metabolites stored in inactive form in subcellular compartments, mostly vacuoles, which become spontaneously and/or enzymatically active upon cell damage (‘ignition’). Finally, metabolites can also be synthesized upon stimulation, being classified as phytoalexins. However, often a single specialized metabolite may fall between these two categories, having a dominant but not absolute production fingerprint.

Elicitation is a most relevant environmental mediator of accumulation of natural products in the phytoalexin spectrum, often improving yields of economically targeted metabolites (Hassini *et al*. 2019; Thakur *et al*. 2019). Elicitors are chemical compounds or physical variables that induce morphological and physiological changes, notably in plant defence profiles, often promoting accumulation of specialized metabolites to protect cells, tissues, organs, and the whole plant (Junkes *et al*. 2019).

The medicinal tree *Erythrina velutina* (Fabaceae) grows in the drylands of northeastern Brazil (Caatinga). This exclusively Brazilian biome is one of the hottest dry regions in the world, with mean temperatures from 25 to 30°C and irregularly distributed rainfall of 600 to 1000 mm in most of its parts (Tabarelli *et al*. 2018). The rainy season is short, lasting 3 to 5 months (January to May), sometimes characterized by flash floods, although droughts can last several years. In addition, almost all the water bodies within the region are transient. During the typical 7 to 9 months of the dry season, soil temperature can reach 60°C (EMBRAPA 2022). Most Caatinga soils are rocky and shallow, with little water retention capacity. Solar irradiance is high, ca. 2800 h year^−1^. Despite the harshness of edaphoclimatic features, Caatinga is a highly biodiverse biome (Tabarelli *et al*. 2018).

*Erythrina velutina* is notable for its exceptional capacity to produce bioactive natural compounds, such as alkaloids and flavonoids. These specialized metabolites are chemical markers and pharmacological agents, with anti‐inflammatory and central nervous system modulating activities (Fahmy *et al*. 2018, 2020; Adetunji *et al*. 2024). Alkaloids and flavonoids have diverse functions in plant growth and development, including involvement in signalling pathways and defence against biotic or abiotic factors (Yonekura‐Sakakibara *et al*. 2019; Acharjee *et al*. 2022). Because of their significant medicinal potential, there is growing interest in increasing yields by elicitation, mimicking environmental signals that can modulate specialized metabolite biosynthesis. Furthermore, such knowledge will assist in the sustainable use of *E. velutina* as a source of alkaloids and flavonoids, as well as providing phytochemical information to improve plant survival under climate extremes.

To date, most research on *E. velutina* has centered on its therapeutic potential. Although some studies have examined seed viability and seedling growth under stress (Ribeiro *et al*. 2017; Lopes *et al*. 2019; Souza *et al*. 2020; Aderaldo *et al*. 2022), research is needed into the role of environmental and phytohormonal elicitation treatments on *E. velutina* as modulators of its chemistry. In our previous research, we produced the first transcriptome analysis of *E. velutina* leaves and seeds, as well as proteome and metabolite profiling in field‐grown natural populations. These studies revealed key targets in biosynthesis of alkaloids and flavonoids, as well as signalling factors related to stresses, such as temperature, radiation (visible and ultraviolet), salt, water deprivation, and phytohormones (Chacon *et al*. 2021, 2022).

Based on these initial findings, laboratory experiments were conducted to investigate the impact of these stress factors and their signalling molecules on metabolite profile and yield. To that end, fingerprint analysis was employed. This is a powerful technique that enables non‐targeted analysis of metabolic composition of an organism. In this method, quantification is relative between control and treated samples, providing classification and/or group information related to metabolites in response to different experimental conditions (Pilon *et al*. 2020). The impacts of four stress‐signalling phytohormones (methyl jasmonate, salicylic acid, nitric oxide, and abscisic acid) and five environmental stresses (temperature, ultraviolet radiation, drought, salinity, and mechanical damage) on the specialized metabolic profile of *E. velutina* seedlings were examined. Leaves and roots were collected at different times after a period under stress (heat, ultraviolet radiation, drought) or after a single initial exposure event (mechanical damage, salinity, phytohormones) and analysed by HPLC‐DAD to identify main differences in chemical profile between experimental conditions. Both unique and shared metabolic responses were observed, often in an organ‐dependent fashion, providing insights into spatiotemporal regulation of *E. velutina* secondary metabolites. This research constitutes a key step to understand the regulation dynamics of specialized metabolism in this highly resilient tree species.

## METHODS

### General experimental procedures

A Waters Alliance Series HPLC system was employed to generate chromatographic profiles. The analysis utilized an NST C18 reverse‐phase column (250 mm × 4.6 mm × 5 μm). Phytohormones were sourced from Sigma‐Aldrich (St. Louis, MO, USA), whereas sodium nitroprusside and sodium chloride were obtained from Vetec Fine Chemicals (Duque de Caxias, RJ, Brazil).

### Plant material and growing conditions

*Erythrina velutina* seeds were obtained from four natural populations in the state of Rio Grande do Norte, Brazil, in the Caatinga biome (cities of Acari and Jardim do Seridó), as well as the National Forestry Conservation Unit (city of Nísia Floresta). Seeds were surface sterilized and subsequently germinated in plastic pots of 600 mL filled with vermiculite:sand (1:1, v/v), kept in a growth chamber at 24 ± 2°C, under a 12‐h photoperiod, irradiance of 65 μmol m^−2^ s^−1^ provided by fluorescent white lights, and irrigation to pot capacity unless stated otherwise (Scheme S1). Authorization to harvest plant material was granted by SISBIO (327493) and access to the Brazilian genetic heritage was provided by SISGEN (A8E4663).

### Experimental design and elicitors treatments

**Fig. 1:**
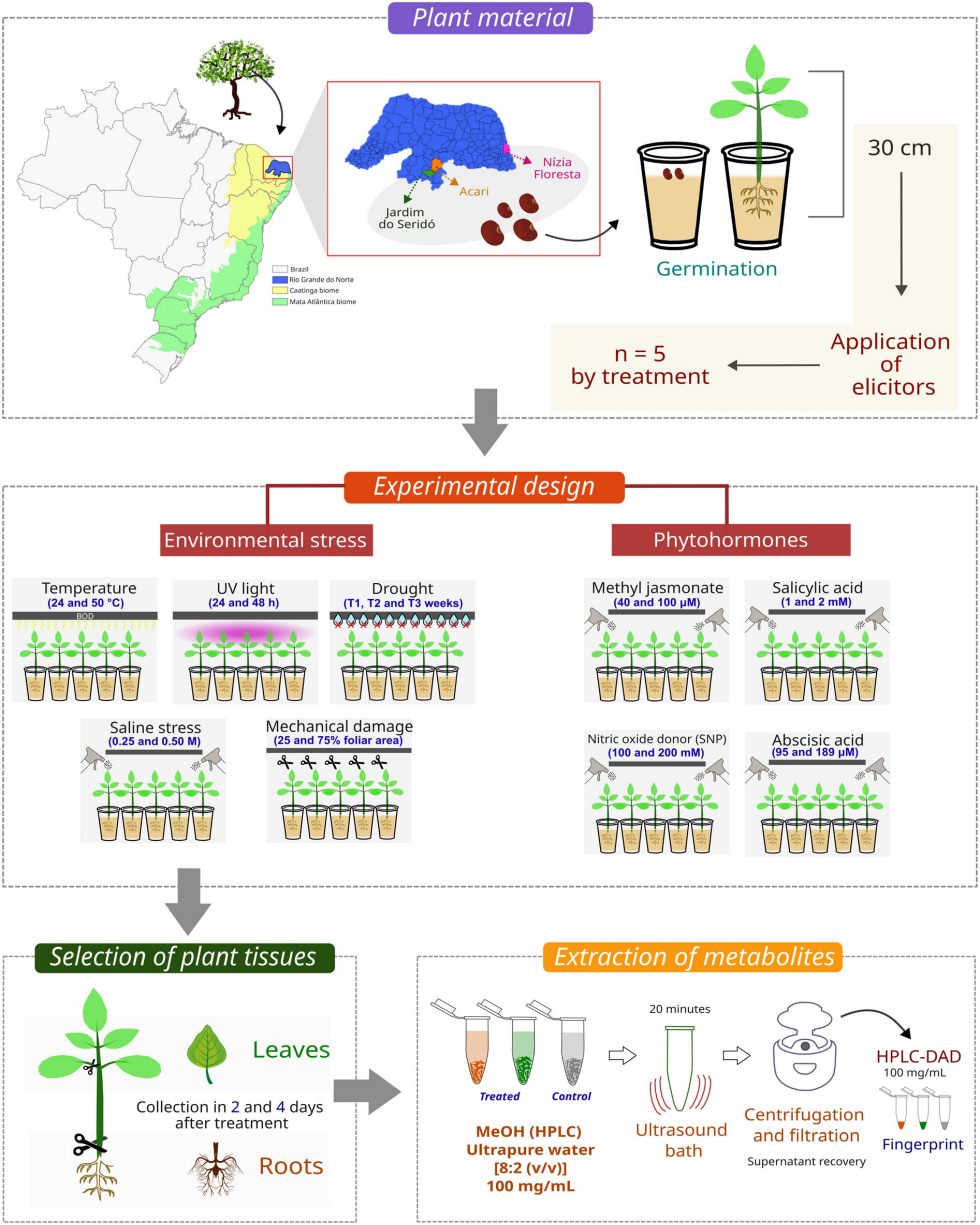
Experimental design and fingerprint analysis methodology in *Erythrina velutina*. The figure illustrates the experimental design and analytical methodology used for fingerprint analysis. Plant samples were treated with different environmental stressors and phytohormones, followed by extraction and analysis using chromatography techniques. The resulting chromatograms were used to generate a fingerprint of the leaf and root samples of the plants (for details of replicates in each group, see supplementary figures and Table S1).

The experiment used healthy seedlings, ca. 30 cm in height 1 month after sowing. Seedlings were randomly assigned to groups of five individuals (biological replicates) then subjected to nine different treatments: five related to exposure to environmental stress and four related to phytohormone application (Fig. 1). Each treated group had a corresponding control with the same number of replicates.

Environmental treatments were: (a) exposure to high temperature for 6 h (24°C versus 50°C) with a single harvest after the exposure period; (b) acute high energy UV light exposure for 24 and 48 h (UV‐C Germicidal Lamp OSRAM, HSN 15 W G13, 90 V ‐ São Paulo, Brazil, about 40 cm above the seedlings) with harvests at the same times; (c) water limitation (irrigation suspended for 1, 2, or 3 weeks) with harvests at the end of each week; (d) mechanical damage (cutting off 25% or 75% of total leaf area) at the start of the experiment, with harvests after 2 and 4 days; (e) salinity stress induced by spraying with sodium chloride (NaCl 0.25 M or 0.50 M) solution at the beginning of the experiment and harvests after 2 and 4 days. Phytohormone exposure treatments consisted of spraying aqueous solutions of methyl jasmonate (40 μM or 100 μM), salicylic acid (1 mM or 2 mM), sodium nitroprusside – a nitric oxide generator (100 mM or 200 mM), or abscisic acid (95 μM or 189 μM). All phytohormone treatments were applied only at the start of experiments, and harvests were done after 2 and 4 days.

Spraying was stopped at the point of imminent run off. The same procedure was used for control seedlings sprayed with water. For all treatments, leaves and roots of seedlings were separately harvested, dried in an oven at 40°C for 24 h, and protected from light for about 1 month prior to metabolite extraction. The experimental scheme is provided in Table S1.

### Extraction of metabolites

The dried biomass was weighed (25 mg) in 2 mL plastic microcentrifuge tubes and pulverized in liquid nitrogen with a microtube pestle. The powder was then extracted with HPLC‐grade methanol and ultrapure water (UPW) (80:20, v/v) at 100 mg mL^−1^, followed by 20 min in an ultrasound bath (Ecosonics, 40 kHz, 200 W) at room temperature. Samples were centrifuged for 2 min (16,000 × *g*, room temperature) to obtain supernatants, which were filtered through 0.22 μM H‐PTFE syringe filters (diameter 13 mm) before injection into the chromatography system for HPLC‐DAD analysis (Fig. 1). Gallic acid (Sigma‐Aldrich) was used as internal standard at 40 μg mL^−1^ during preparation of the extraction solution (MeOH and water [80:20, v/v]). This had a retention time of ca. 10 min.

### HPLC‐DAD analysis

The hydroalcoholic extracts were injected into a Waters Alliance series HPLC system with degasser, automatic injection, and a diode array detector. For each sample, 7 μL were injected into an NST C18 reverse phase column (250 mm × 4.6 mm × 5 μm). The elution mode was a gradient, composed of water +0.1% trifluoroacetic acid (phase A) and acetonitrile +0.08% trifluoroacetic acid (phase B). Composition of the gradient was optimized: 100% phase A (0–15 min), 65% phase A and 35% phase B (15–23 min), 35% phase A and 65% phase B (23–40 min), and 100% phase B (40–50 min) (Table S2). After 50 min, the column was equilibrated for 10 min with 100% phase A to return to the starting condition and injection of the next replicate. The mobile phase flow rate was 0.8 mL min^−1^, and DAD spectra were recorded between 220 and 400 nm. After every five biological replicates, a blank containing only MeOH (HPLC grade) and ultrapure water (80:20, v/v) was injected at the same injection volume as the samples.

A mixture of known flavonoids and alkaloids was analysed for comparison with chromatographic profiles of the treatments, enabling estimation of retention times where these compounds – flavonoids and alkaloids – eluted in the treated samples (Table S3). The isoquinoline alkaloids (erythraline, isomers erysodine and erysovine, lycorine, montanine, and tazetine) and flavonoids (7,4‐hydroxyflavone, daidzein, quercetin, genistein, kaempferol, and formononetin) were analysed at 1 mg mL^−1^ in methanol:ultra‐pure water (80:20, v/v). The standards 7,4‐hydroxyflavone, quercetin, and kaempferol were purchased from Sigma‐Aldrich, whereas other compounds were isolated by our group and deposited in the laboratory chemical library as reference substances (de Andrade *et al*. 2012; Guaratini *et al*. 2014; Tallini *et al*. 2017; Reis *et al*. 2019a, 2019b; de Almeida *et al*. 2021).

### Statistical analysis

#### Chemoinformatic analysis

The raw chromatogram absorbance files (.arw) were downloaded and corrected by subtracting absorbance values of the corresponding blank samples. The chromatograms were aligned within each experimental group, comparing treated samples to their respective control groups. Only chromatograms that had improved alignment without introducing noise were kept in the final analysis. For chromatograms where alignment did not improve or resulted in additional noise, the original chromatograms (unaligned) were used (Table S4). Alignment used the iCoshift algorithm (Savorani *et al*. 2010; Tomasi *et al*. 2011) that enabled alignment of the whole chromatogram or at intervals. The best visual alignment was selected for the chemoinformatic pattern recognition analysis.

Following alignment, normalization was performed using gallic acid as internal standard, using the following equation:

(1) $$C_{\mathrm{nor}}\;=\;\frac{C_{\mathrm{ori}}}{S_{\mathrm{obs}}}\;S_{\exp}$$

where, *C*_nor_ is height of peaks in the corrected chromatogram, *C*_ori_ is height of peaks in the uncorrected original chromatogram, *S*_obs_ is height of peak of the internal standard in the original chromatogram, and *S*_exp_ = 0.0942 (absorption units) is expected height of the peak of the internal standard. Equation (1) corrects for minor variations in the internal standard across samples by adjusting peak heights to match the expected standard value.

Leaf and root profiles of *E. velutina* were evaluated by analysing chromatograms at 280 nm. An initial exploratory analysis was performed to assess relative quantification of seedling responses to environmental factors and phytohormones. This analysis was based on differences in absorbance between each treated chromatogram and its corresponding control. Both analysis and chromatogram plots were generated using Python, specifically the pandas (https://pypi.org/project/pandas/), numpy (https://pypi.org/project/numpy/), and matplotlib (https://matplotlib.org/) packages.

A fold change (FC) analysis was performed, highlighting peaks with FC >1.5, indicating they were >50% higher in the treated compared to the control group. Peaks that exceeded average absorbance value of chromatograms from both treated and control groups were selected for the fold change analysis. This average absorbance served as a threshold for peak selection.

To assess significant differences in peak intensities between control and treatment groups, peaks with FC ≥1.5 were selected for statistical analysis. For each selected peak, maximum intensity values from biological replicates were tested for normality using the Shapiro–Wilk test and for homogeneity of variances using Levene's test. When both assumptions were met (*P* > 0.05), a Student's *t*‐test was performed, and effect size estimated using Cohen's *d*. If both assumptions had *P* < 0.05, a non‐parametric Mann–Whitney *U* test was applied, with effect size calculated using the r statistic. Statistical significance was defined as *P* < 0.05. All analyses were performed in Python using the scipy.stats module for statistical testing and the pandas package for data handling.

Following the same analytical workflow, a complementary analysis was performed to evaluate peaks in sample chromatograms with retention times similar to those of some injected chemical standards (isomers erysodine‐erysovine, erythraline and quercetin) under identical HPLC conditions. For these matched peaks, the area under the curve was calculated using Simpson's composite rule, and statistical analyses were performed using the same procedures as for peak intensity data.

For the multivariate method, ANOVA of the principal components analysis (ANOVA‐PCA) was performed using an algorithm in the MATLAB environment, v. R2010a, based on Harrington *et al*. (2005). Information on the implemented algorithm is in the supplementary material of Herrera *et al*. (2022). Data pre‐processing and PCA for ANOVA‐PCA used a graphical user interface in the R environment (Darzé *et al*. 2022, 2023). The number of principal components was determined using a scree plot and selected components based on accumulated variance for subsequent score and loading analysis. The confidence limits calculated with a Hotelling T2 test were plotted for each metabolic stress at score plot to identify significant differences. Additionally, the Hotelling T2 vs. residual Q test was used to identify anomalous samples.

## RESULTS

### Determination of retention time range of *Erythrina* metabolite classes: alkaloids and flavonoids

HPLC‐DAD was used to analyse the chemical profile of leaves and roots of *E. velutina* after treatment with elicitors to identify similarities, differences, and overall metabolite accumulation in this species. A detailed chromatographic profile for the different treatments is presented, focusing on differences between environmental factors and phytohormones. HPLC‐DAD is a valuable analytical tool for comparison of several samples and metabolic fingerprints. Herein, mapping of major differences in chemical responses of *E. velutina* treated with several elicitors (environmental stresses and phytohormones) was developed using statistically accurate tools to support future in‐depth studies of structural assignments and involved biosynthetic pathways.

Recognizing that molecular identification is one limitation of HPLC‐DAD, this approach was used to analyse a pool of standards for flavonoids and alkaloids previously isolated in our laboratory, as well as other isoquinoline alkaloids structurally related to those in *Erythrina* spp. (Table S3). This approach allowed establishment of chemical class distributions throughout the chromatogram. Profiles revealed that alkaloids typically eluted in the mid‐portion of the chromatogram (10 min < RT < 20 min), whereas flavonoids eluted at the end (RT 20 min; Fig. S1). This is congruent with our previous studies for structural annotation (Chacon *et al*. 2021, 2022) and indicated that our approach is likely effective in capturing chemical diversity within the samples. Furthermore, both alkaloid and flavonoid classes may have been altered in the treated groups compared to the controls.

### Effects of environmental stress factors on metabolite production

#### Temperature

**Fig. 2:**
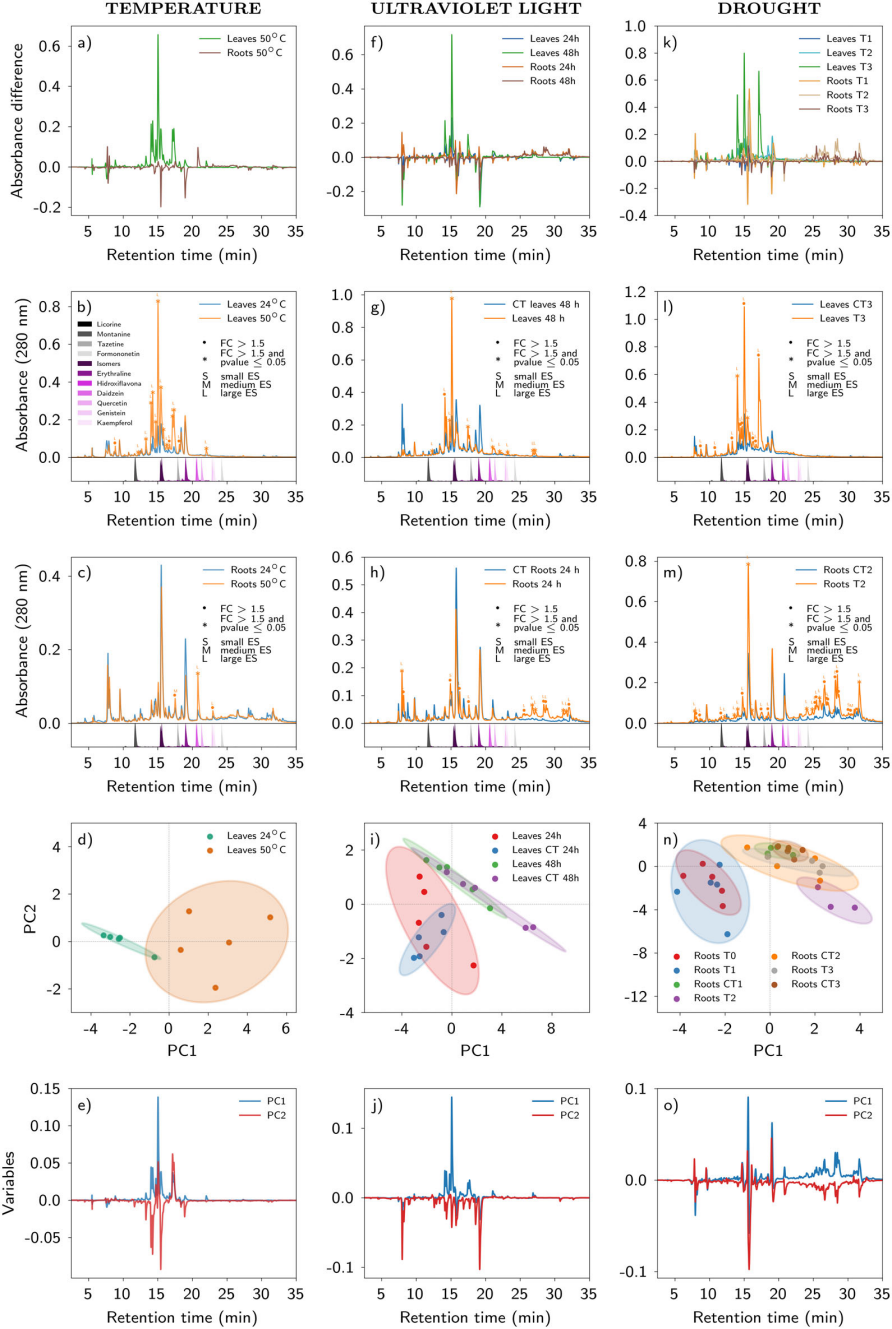
Relative quantification and responses of the metabolic profile in *Erythrina velutina* seedlings exposed to temperature, UV light, or drought treatments. Panels are grouped vertically in columns of three, with each column representing a specific treatment: temperature, ultraviolet light, or drought. In Panels (a, f, k), the Y‐axis represents the absorbance difference at 280 nm between each treated plant chromatogram and its respective control. Peaks with an absorbance difference >0 indicate an increase in the treated group, whereas peaks with an absorbance difference <0 indicate a decrease in the treated group. The X‐axis represents chromatography retention time. Panels (b, c, g, h, l, m) show the conditions that led to the highest or one of the highest compound accumulations in leaves and roots for each respective treatment. A cut was applied at 35 min to aid visualization of the peaks. Each chromatogram is the average of biological replicates (for details of replicates in each group, see supplementary figures and Table S1). •Coloured circles indicate peaks with a fold change (FC) >1.5, with each colour representing a corresponding experimental group. *Coloured asterisks indicate peaks with FC >1.5 and *P*‐value < 0.05, with each colour representing a corresponding experimental group. Effect size analysis was performed, and results are indicated in the legend as S (small), M (medium), or L (large). These classifications indicate the magnitude of differences between groups and support interpretation of results. A medium or large effect size suggests biologically relevant differences, even when the *P*‐value is not statistically significant. Peaks marked with a circle or asterisk but without an effect size label indicate no significant effect was observed. Coloured peaks in the lower part of the chromatogram represent injected standards of alkaloids and flavonoids, with purple indicating compounds previously identified in the *Erythrina* genus. Panels (d, i, and n) show the PCA score plots, while Figures (e, j, and o) display the corresponding loading plots, all derived from the PCA that provided the best group separation. In the score plots, ellipses represent 95% confidence intervals for each class. Principal components are distinguished by different colours in loading plots. A retention time threshold of 35 min was applied to focus the analysis on the main peaks.

Heat shock of 50°C for 6 h led to accumulation of several major metabolites in leaves, while there was an opposite trend in roots (Fig. 2a). Specifically, in leaves multiple peaks with higher absorbances were detected at 50°C compared to treatment at 24°C, with highest accumulations in the mid‐portion of the chromatogram run (14 min < RT < 20 min; Fig. 2b). This represented the experimental group with the highest compound intensities under heat shock (i.e., several peaks with FC >1.5, *P*‐value <0.05, and large effect size). In contrast, at 50°C, roots accumulated fewer peaks that were also less intense (Fig. 2c).

The ANOVA‐PCA also supported these findings, revealing partial separations between leaves at 24°C and at 50°C. PC1 values were mainly positive, indicating higher accumulation in leaves at 50°C (Fig. 2d,e). Despite differences in chemical profiles between the organs, there were some peaks with similar retention times in leaves and in roots (Fig. 2b,c).

#### Ultraviolet light

Exposure of *E. velutina* seedlings to high energy UV light led to accumulation of metabolites in leaves and roots (Fig. 2f). Leaves had higher accumulation across several peaks compared to the control at both time points, mainly after 48 h, while root accumulation was more pronounced after 24 h (Fig. 2f, Fig. S4).

Leaves exposed to UV for 24 h had three peaks in the mid‐portion of the chromatogram (14 min < RT < 16 min) that were pronounced (Fig. S4a,b), with accumulation increasing after 48 h of exposure (FC >1.5, two peaks with *P*‐value < 0.05; Fig. 2g). Some peaks accumulated in roots after 24 h of UV exposure (Fig. 2h), however, their levels decreased after 48 h (Fig. S4d). Moreover, exposure to UV light for 24 and 48 h mainly led to appearance of novel compounds in roots, with several peaks FC >1.5, *P*‐value < 0.05 and large effect size (Fig. 2h). These peaks emerged towards the end of the chromatographic run (25 min < RT < 35 min). Overall, leaves exposed to UV light for 48 h had the highest accumulation and intensity of compounds in this treatment (Fig. 2g). ANOVA‐PCA showed partial separation in leaf samples between the 24 and 48 h, highlighting differences in profiles at the two time intervals (Fig. 2i,j).

#### Drought

Water deprivation resulted in metabolite accumulation in both roots and leaves. Monitoring peaks at 280 nm and analysing chromatographic profiles revealed differences in accumulation between the two organs (Fig. 2k). After 1 week of water limitation, leaves showed discrete accumulation of peaks compared to the constantly irrigated control (CT1) and control at the start of the experiment (T0) (Fig. S6a,b). After 2 weeks of drought, this accumulation increased across all peaks compared to the 2‐week control (CT2) (Fig. S6c), although leaf total peak intensity was not higher than that after 1 week. In week 3, there were important accumulations (FC >1.5; *P*‐value < 0.05) of ca. four‐fold of initial levels (1 and 2 weeks) in several chromatogram peaks of seedlings under water deprivation compared to the corresponding controls (CT3 and CT0) (Fig. 2l, Fig. S6e,f).

Root chemical profiles were distinct from those of leaves. During week 1 of water limitation, some peaks accumulated in leaves, along with the discrete appearance of new peaks in roots, particularly at 25 min < RT < 35 min (Fig. S7a). In week 2 of drought, peaks in roots became more prominent, with absorbance higher than that after week 1 of water limitation (Fig. 2m, Fig. S7c). Similarly, peaks in the final portion of the chromatogram (25 min < RT < 35 min) accumulated to higher levels (Fig. 2m). During week 3 of water limitation, some peaks in roots had lower intensity compared with weeks 1 and 2 (Fig. S7e,f). The ANOVA‐PCA essentially confirmed the described metabolite accumulation profiles (Fig. 2n,o). There was a partial but considerable separation between control and treated roots after 1 and 2 weeks of water stress.

#### Salt stress

**Fig. 3:**
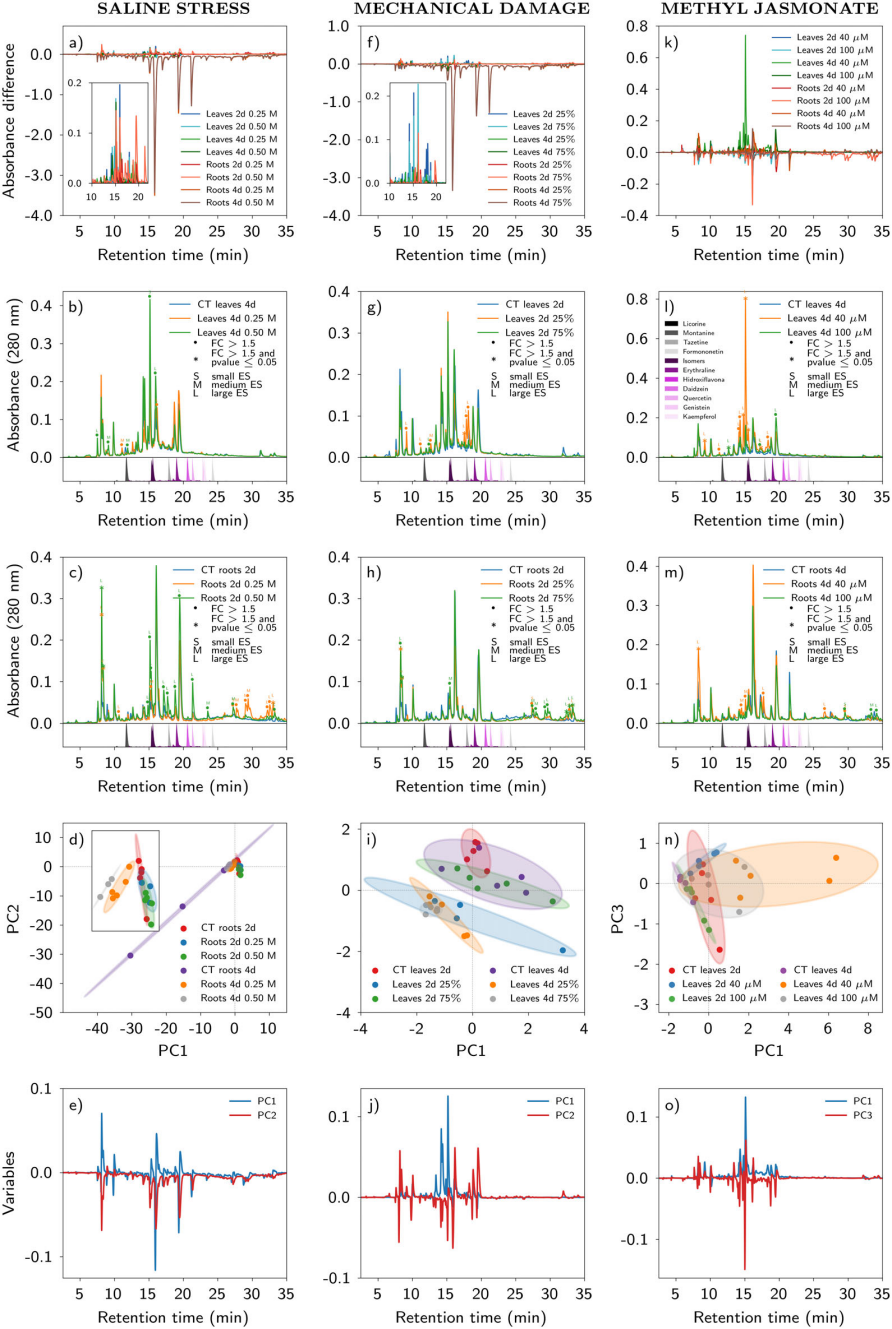
Relative quantification and responses of the metabolic profile in *Erythrina velutina* seedlings exposed to saline stress, mechanical damage, or methyl jasmonate treatment. Panels are grouped vertically in columns of five, with each column representing a specific treatment: saline stress, mechanical damage, or methyl jasmonate. In Panels (a, f, k), the Y‐axis represents the absorbance difference at 280 nm between each treated plant chromatogram and its respective control. Peaks with an absorbance difference >0 indicate an increase in the treated group, whereas peaks with an absorbance difference <0 indicate a decrease in the treated group. The X‐axis represents chromatography retention time. Panels (b, c, g, h, l, m) show conditions that led to the highest, or one of the highest, compound accumulations in leaves and roots for each respective treatment. A cut was applied at 35 min to aid visualization of the peaks. Each chromatogram is the average of biological replicates (for details of replicates in each group, see supplementary figures and Table S1). •Coloured circles indicate peaks with a fold change (FC) >1.5, with each colour representing a corresponding experimental group. *Coloured asterisks indicate peaks with FC >1.5 and *P*‐value < 0.05, with each colour representing a corresponding experimental group. Effect size analysis was performed, and results are indicated in the figure legend as S (small), M (medium), or L (large). These classifications indicate the magnitude of differences between groups and support interpretation of the results. A medium or large effect size suggests biologically relevant differences, even when the *P*‐value is not statistically significant. Peaks marked with a circle or asterisk but without an effect size label indicate no significant effect was observed. The coloured peaks in the lower part of the chromatogram represent injected standard of alkaloids and flavonoids, with purple indicating compounds previously identified in the *Erythrina* genus. Panels (d, i, and n) show PCA score plots, while Figs (e, j, and o) display the corresponding loading plots, all derived from the PCA that provided the best group separation. In the score plots, ellipses represent 95% confidence intervals for each class. Principal components are distinguished by different colours in loading plots. A retention time threshold of 35 min was applied to focus analysis on the main peaks.

Salinity affected metabolite profiles in leaves and roots, although less intensely than temperature, UV, or water limitation (Fig. 3a). The impact of salinity on metabolite profiles varied with concentration and time after application. Leaf exposure to NaCl after 2 and 4 days resulted in accumulation of different metabolites at 0.25 M and 0.50 M (Fig. 3b, Fig. S9a,b). Some peaks increased more on different days depending on salt concentration. However, few of the accumulated peaks had a fold change (FC) >1.5 (Fig. S9a,b).

In roots, application of 0.50 M NaCl resulted in accumulation of several main peaks with FC >1.5 after 2 days. Even though peaks had *P*‐value > 0.05, they had a substantial overall effect size, suggesting potential biological relevance (Fig. 3c). Indeed, this treatment led to some of the most pronounced accumulations. Two days after the treatment, chromatograms (RT > 25 min) showed new peaks began to accumulate in roots, particularly with 0.25 M NaCl (Fig. 3c). After 4 days of salt application, there was an important decrease in number of peaks in roots treated with 0.50 M or 0.25 M NaCl compared to controls (Fig. S9d), although peak intensity of this chromatogram was highest. Two days after the exposure of roots, the largest accumulation differences were observed (Fig. 3c), particularly with 0.50 M NaCl. Additionally, the ANOVA‐PCA showed notable separation between 2‐day and 4‐day root samples along PC1, with positive PC1 peaks more abundant at 2 days (Fig. 3d,e).

#### Mechanical damage

Similar to salt stress, mechanical damage modified metabolite accumulation in leaves and roots of seedlings, but the effect was less pronounced compared to temperature, UV, or water limitation (Fig. 3f). In leaves, larger increases in peak absorption with 25% or 75% damage were recorded after 2 days compared to 4 days (Fig. 3g, Fig. S11a,b). Furthermore, leaves 2 days after wounding had more peaks with FC >1.5, mainly following 25% mechanical damage (Fig. 3g). After 4 days of the mechanical stress infliction, peak intensity generally decreased in both control and treated leaf samples (Fig. S11b).

Chemical profiles in roots 2 days after leaf mechanical damage were also affected. Most of the accumulated peaks were after intense leaf damage, i.e., 75% of the foliar area (Fig. 3h). At the end of the analysis, several root peaks accumulated 2 days after damage, but most were absent in control samples (Fig. 3h). Leaves subjected to mechanical damage had greater differences in compound accumulation (inset in Fig. 3f), particularly after 2 days (Fig. 3f). The ANOVA‐PCA revealed partial separation between the 2‐day and 4‐day time points in both leaves and roots (Fig. 3i,j).

### Effects of phytohormones on the production of metabolites

#### Methyl jasmonate (MeJA)

Application of MeJA to *E. velutina* seedlings resulted in accumulation of several metabolites in leaves and roots, particularly after 40 μM MeJA, mainly in the roots (Fig. 3k). In leaves, 2 days after 40 μM MeJA exposure produced the most prominent peaks; however, some peaks were smaller than those in the 2‐day control (Fig. S13a). At 4 days, nearly all peaks increased after MeJA application, many with FC >1.5 and a significant *P*‐value, particularly with 40 μM MeJA (Fig. 3l). Exposure to 40 μM MeJA strongly induced accumulation of metabolites.

In roots, most peaks also accumulated 4 days after application of 40 μM MeJA (Fig. 3m). At the end of the chromatographic run, some peaks accumulated more in control samples after 2 days (Fig. S13c).

The leaf ANOVA‐PCA gave better partial separation from the control at 4 days after treatment than that for roots and 40 μM MeJA at the same time point (Fig. S14a,c). The treated group had peaks with a positive PC1, indicating peak abundance along this component (Fig. 3n,o).

#### Salicylic acid (SA)

**Fig. 4:**
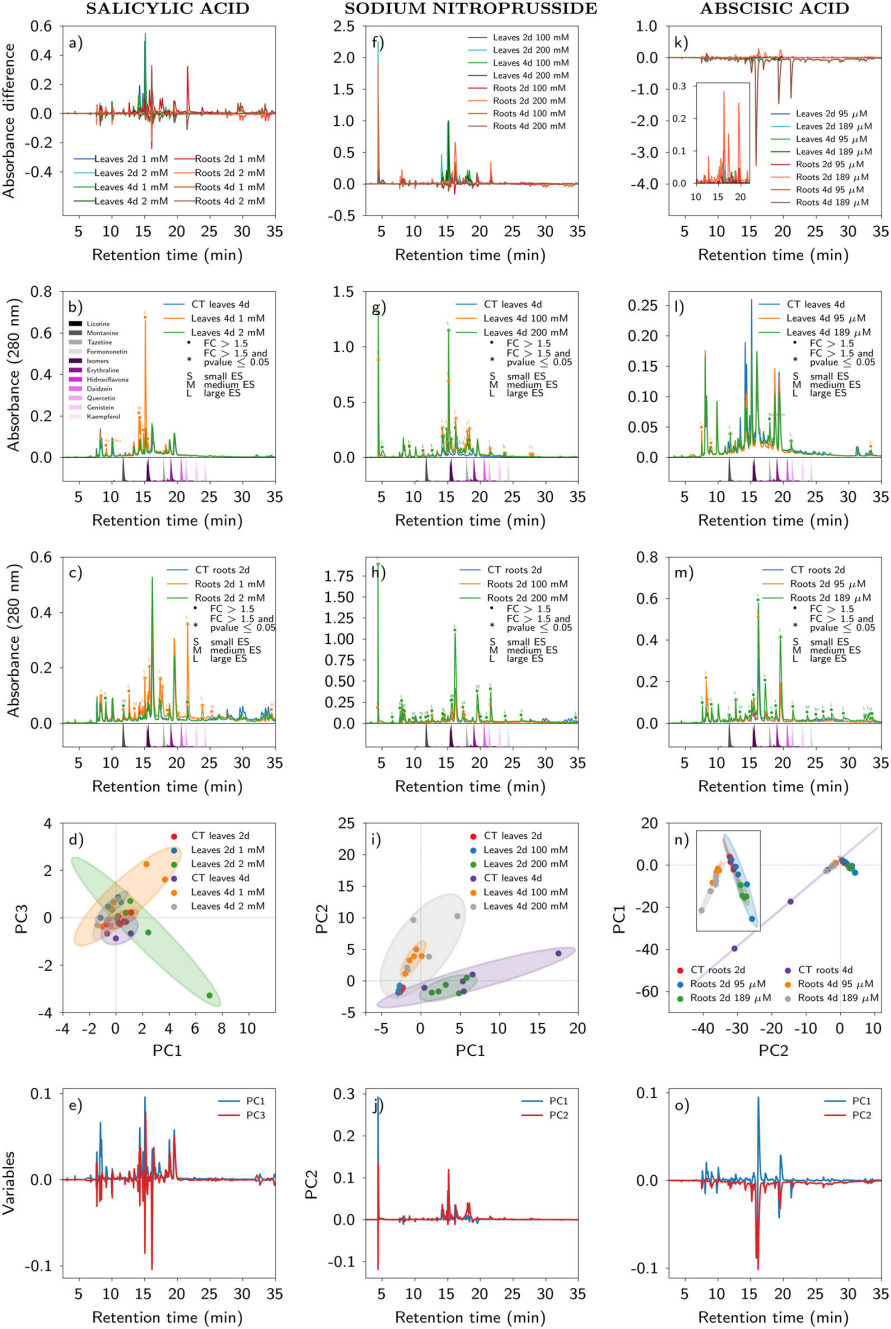
Relative quantification and responses of the metabolic profile in *Erythrina velutina* seedlings exposed to salicylic acid, sodium nitroprusside or abscisic acid. Panels are grouped vertically in columns of five, with each column representing a specific treatment: salicylic acid, sodium nitroprusside or abscisic acid. In Panels (a, f, k), the Y‐axis represents absorbance difference at 280 nm between each treated plant chromatogram and its respective control. Peaks with an absorbance difference >0 indicate an increase in the treated group, whereas peaks with an absorbance difference <0 indicate a decrease in the treated group. X‐axis represents chromatography retention time. Panels (b, c, g, h, l, m) show conditions that led to the highest or one of the highest compound accumulations in leaves and roots for each respective treatment. A cut was applied at 35 min to aid visualization of the peaks. Each chromatogram is average of biological replicates (for details of replicates in each group, see supplementary figures and Table S1). •Coloured circles indicate peaks with a fold change (FC) >1.5, with each colour representing a corresponding experimental group. *Coloured asterisks indicate peaks with FC >1.5 and *P*‐value < 0.05, with each colour representing a corresponding experimental group. Effect size analysis was performed, and results are indicated in the figure legend as S (small), M (medium), or L (large). These classifications indicate magnitude of the differences between groups and support interpretation of the results. A medium or large effect size suggests biologically relevant differences, even when the *P*‐value is not statistically significant. Peaks marked with a circle or asterisk but without an effect size label indicate no significant effect observed. Coloured peaks in the lower part of the chromatogram represent injected standard of alkaloids and flavonoids, with purple indicating compounds previously identified in the *Erythrina* genus. Panels (d, i, and n) show PCA score plots, while Figures (e, j, and o) display corresponding loading plots, all derived from the PCA that provided the best group separation. In score plots, ellipses represent 95% confidence intervals for each class. Principal components are distinguished by different colours in loading plots. A retention time threshold of 35 min was applied to focus analysis on the main peaks.

Application of SA to *E. velutina* revealed that 1 mM was more effective in promoting metabolite accumulation, both 2 and 4 days after spraying, especially in leaves (Fig. 4a). In leaves, after 2 days, several peaks with FC >1.5 accumulated upon exposure to 1 mM SA (Fig. S15a). After 4 days, these major peaks continued to accumulate compared to the control (Fig. 4b). In roots, most peaks (RT < 25 min) accumulated after 2 days of treatment, either at 1 mM or 2 mM SA, but more so at the lower concentration, with FC >1.5 and *P*‐value < 0.05 (Fig. 4c). At 4 days, the number of peaks after 1 mM SA application fell significantly, with little fold change at 2 mM SA (*P*‐value in RT > 25 min; Fig. S15d).

Although not totally separated, leaf ANOVA‐PCA showed that control samples at 2 and 4 days had negative scores on PC3, while samples treated with 1 mM SA after 2 or 4 days showed the opposite (Fig. 4d). This indicates accumulation of peaks in the positive PC3 on the loadings plot for the treated groups (Fig. 4e). Notably, the 4‐day post‐treatment time produced the most important differences in peak accumulation compared to its respective control, and reached highest absorbance in the chromatographic analyses (Fig. S15).

#### Sodium nitroprusside (SNP)—source of nitric oxide (NO)

Application of SNP to *E. velutina* seedlings induced metabolite accumulation at both 100 mM and 200 mM SNP (Fig. 4f). The 200 mM SNP treatment after 2 and 4 days resulted in accumulation of practically all leaf peaks with FC >1.5, *P*‐value < 0.05, and large effect size (Fig. 4g). These conditions resulted in higher peak accumulations in the treated group compared to the corresponding controls (Fig. S17a,b). Furthermore, one peak showed higher accumulation at the start of the chromatographic run (<5 min), was four times more abundant than the average of any other peak, and was not present in control samples (Fig. 4f, Fig. S17).

After 2 days, treated roots accumulated various peaks at 200 mM SNP (Fig. 4h) but these decreased after 4 days (Fig. S17c,d). Overall, peaks in leaves were more intense at both 2 and 4 days after SNP application compared to roots (Fig. S17). The ANOVA‐PCA revealed a notable separation in leaf samples after 4 days: the control group had negative scores on PC2, whereas samples treated with 100 or 200 mM SNP had positive scores on PC2 (Fig. 4i,j), despite not achieving total separation.

#### Abscisic acid (ABA)

The impact of ABA treatment at 95 μM or 189 μM on accumulation of metabolites in *E. velutina* leaves and roots was the least effective in affecting metabolite profiles among phytohormones (Fig. 4k). In leaves, after 2 days of the spraying treatment, control samples had higher intensity of various major peaks (Fig. S19a). After 4 days of ABA application, the number of intense peaks (FC >1.5 and significant *P*‐value) compared to control samples slightly increased at 95 μM and 189 μM ABA (Fig. 4l).

In contrast to leaves, roots showed more accumulation of major peaks in treated samples after 2 days, mainly at 189 μM ABA, with most peaks having FC >1.5, *P*‐value < 0.05 and large effect size (Fig. 4m). Although the intensity of both treated and control groups was higher after 4 days compared to 2 days, there was no accumulation in the treated group compared to the control (Fig. S19d). Thus, the most stimulating effect of ABA for peak accumulation was in 2‐day root samples (Fig. 4m, Fig. S19). The ANOVA‐PCA supported this finding, with a notable separation in root samples. Specifically, the 4‐day groups (both control and treated) tended to have negative scores on PC1, while the 2‐day groups (both treated and control) had positive scores on PC1 (Fig. 4n). This suggests accumulation of peaks in the positive region of PC1 for the 2‐day post‐treatment group (Fig. 4o).

## DISCUSSION

*Erythrina velutina*, a medicinal plant traditionally used to treat anxiety, depression, and inflammation, contains natural bioactive compounds, such as alkaloids and flavonoids with therapeutic potential. In previous studies, we described and identified some key targets in biosynthesis of these compounds, as well as signalling pathways that respond to phytohormones and environmental stress using plants harvested in their natural habitat (Caatinga biome). Herein, the focus was on fingerprinting analysis to gain insights into how this plant changes natural products accumulation in response to different stressors applied to specimens cultivated in a greenhouse setting. To that end, HPLC‐DAD analysis was performed on leaves and roots of *E. velutina* seedlings subjected to nine different independent treatments of varying concentrations, exposure, and post‐treatment harvest times. Exposure of *E. velutina* seedlings to different environmental stresses and phytohormones led to changes in metabolite profiles of both leaves and roots. Moreover, effects of some treatments on metabolite accumulation were organ‐, time‐ and/or concentration‐dependent, suggesting fine regulation of specialized metabolite profiles.

**Fig. 5:**
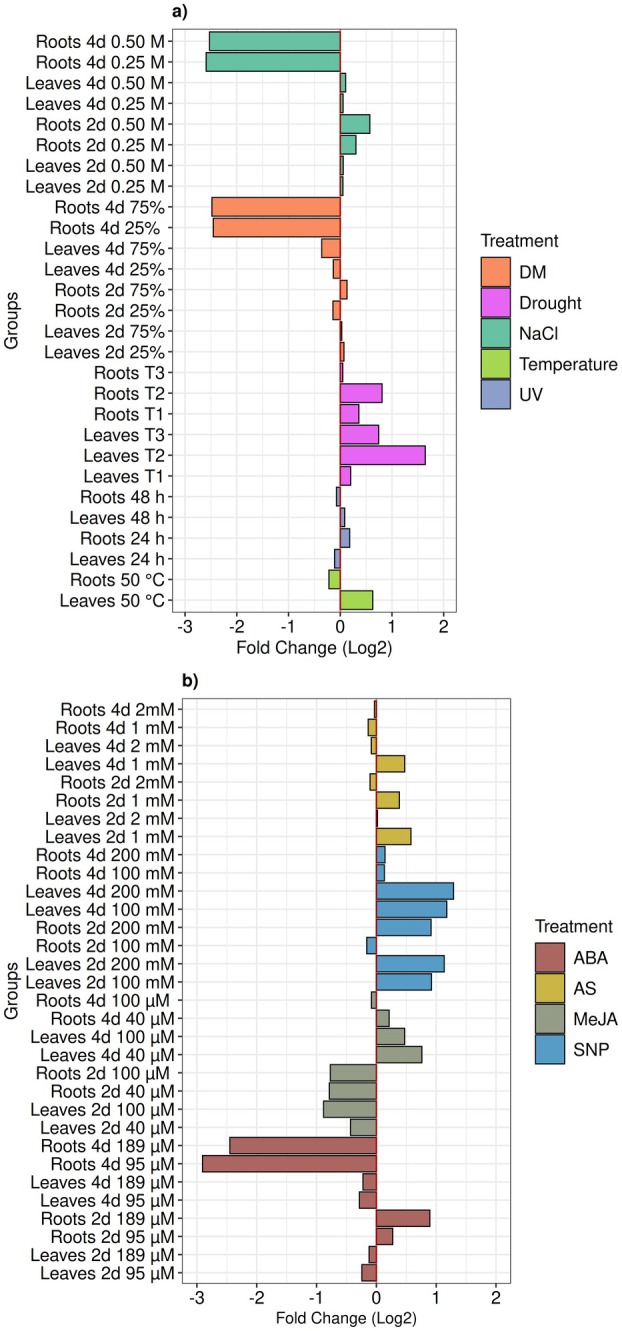
Overview of environmental stress and phytohormone treatments. In (a, b), the X‐axis represents fold change values absorbance (log2), while the Y‐axis denotes experimental groups of environmental factors and phytohormone treatments, respectively. Absorbance profile and area of phytohormone‐treated *Erythrina velutina* seedlings. These data represent fold change of the summed values of all peaks in each chromatogram for each treatment group, compared to their respective controls. This approach was used to provide a comprehensive view of peak accumulation in the treatments.

Initial findings showed that, among the environmental stresses, temperature, UV radiation, and water limitation triggered highest accumulation of metabolites in leaves (Fig. 5a), particularly under more intense treatment conditions. Specifically, extended periods of water restriction (3 weeks), high temperature shock (50°C), or prolonged exposure to acute UV (48 h) resulted in the highest induction of metabolites (Fig. 2). Although all three stressors also induced metabolite accumulation in roots, the most substantial effect was in leaves and after 3 weeks of water deprivation. Interestingly, the Brazilian semi‐arid region, i.e., the Caatinga biome, experiences regular water deficit, uneven rainfall, and high temperature and light intensity, leading to high evaporative demand and soil desiccation (Trovão *et al*. 2007). Remarkably, our findings revealed that the three environmental stresses (temperature, water limitation, and UV radiation) induced several peaks with similar retention times (Fig. S21), suggesting the presence of a shared physiological signature against these stressors in *E. velutina*, which could constitute an adaptation to the Caatinga biome.

In general, roots showed less accumulation of metabolites compared to leaves after high temperature or 48 h of UV exposure (Figs. 2b,c and 5a). Unsurprisingly, UV radiation and high temperatures mainly affected leaves of seedlings, with fewer effects on roots, likely because they are protected underground. Furthermore, plants adapted to hot environments have enhanced mechanisms to deal with thermal stress, maintaining root homeostasis and reducing metabolic costs, thereby conserving carbon sources (Huang *et al*. 2012).

Water limitation led to accumulation of metabolites in *E. velutina* seedlings, with the most pronounced effects after 3 and 2 weeks of stress to leaves and roots, respectively (Fig. S22). Furthermore, roots showed metabolite accumulation after 2 weeks of stress mainly for compounds with RT > 25 min (Fig. S22). This could suggest that the compounds synthesized in the first week are effective in mitigating the effects of water limitation in both roots and leaves, but this effectiveness diminishes after 2 weeks in roots and after 3 weeks in leaves, when an increase in compound accumulation was observed. In leaves after 3 weeks (Fig. S22), with further intensification of water restriction, several peaks accumulated, suggesting that, under long‐term water deprivation, specialized metabolites throughout the plant are deployed as part of a strategy to mitigate dynamic and progressive damage. The accumulation of metabolites, as well their increase in chemical diversity, may contribute to plant survival.

Application of abscisic acid (ABA) and water limitation affected metabolite accumulation. Whereas ABA induced the least accumulation of metabolites, it was still observed that the highest accumulations occurred mainly at higher ABA concentrations (Fig. 4l,m). In leaves, some major peaks observed after 3 weeks of water limitation showed compatible retention times in ABA‐treated leaves at concentrations of 95 or 189 μM ABA after 4 days (Fig. S23a,b). Similar results were observed in roots, with major peaks in water limitation and comparable retention times in ABA‐treated roots (Fig. S23c,d). The observed similarities between the effects of water limitation and ABA probably reflect the known contribution of ABA signalling to water limitation responses by modulating stomatal closure, root growth, activation of gene expression, and metabolic changes (Muhammad Aslam *et al*. 2022). The present findings suggest that ABA may play a role in inducing metabolite accumulation under water restriction, with intensification at higher concentrations and longer treatment times. However, water limitation induced the appearance of new peaks in both leaves and roots, which were not observed after ABA treatment. These results suggest the involvement of ABA‐independent signalling pathways (Muhammad Aslam *et al*. 2022) in mediating metabolic changes that lead to quantitative and qualitative differences in chemical profiles under water limitation.

Methyl jasmonate (MeJA) is widely used to promote production of various phytochemicals in plant cells, organs, and whole individuals (Sohn *et al*. 2022). Spraying plants with the lower concentration of MeJA (40 μM) was more effective in promoting accumulation of metabolites in leaves and roots, particularly in the intermediate portion of the chromatographic run (15 min < RT < 25 min), compared to the higher concentration (100 μM) (Figs. 3l,m and 5b). In agreement with our findings, Bhambhani *et al*. (2012) recorded a 3.7‐fold increase in yield of the alkaloid vasicine in *Adhatoda vasica* cultures treated with 20 μM MeJA, which was not observed at higher concentrations of this phytohormone. On the other hand, Xu *et al*. (2015) found 100 μM MeJa was the optimal concentration for maximizing total stilbene production in *Vitis vinifera*. Clearly, the optimal concentration of MeJA to modulate specialized metabolite accumulation varies, depending on the plant species and experimental system used.

Methyl jasmonate is mostly involved in herbivory responses, with crosstalk through different signalling pathways, such as mechanical damage, abscisic acid (ABA), salicylic acid (SA), and nitric oxide (NO) (Balusamy *et al*. 2015; Ho *et al*. 2020). Herein the effects of MeJA and mechanical damage on *E. velutina* were examined. Whereas MeJA treatment resulted in increased accumulation of metabolites at a lower concentration (40 μM), mechanical damage accumulated metabolites after 25% and mainly 75% loss of foliar area. Interestingly, despite these different response thresholds, peaks in leaves and roots treated with 40 μM MeJA had retention times matching those of peaks in plants subjected to 75% mechanical damage (Fig. S24). A similar situation was observed comparing MeJA with SA treatment. In SA assays, 1 mM was most effective for accumulation of metabolites (Fig. 4b,c). The accumulated peaks of 40 μM MeJA in leaves and roots had retention times compatible with application of 1 mM SA (Fig. S25). This effect was observed in peaks 2 and 4 days after exposure to MeJA and SA (Fig. S25).

The above findings suggest that MeJA, SA, and mechanical damage co‐regulate some metabolites in *E. velutina* in response to stress. Moreover, it appears that relatively lower concentrations of MeJA and SA may be sufficient for signalling to occur compared to the level of mechanical damage needed to trigger similar metabolic changes. However, to verify the interaction between these signalling pathways in *E. velutina*, future studies should include experiments that apply different combinations of MeJA, SA, and mechanical damage, as well use specific phytohormonal pathway inhibitors on the same plants.

Nitric oxide (NO) is a crucial signalling molecule in plant defence responses, with multiple roles, such as regulating oxidative stress as an antioxidant defence, modulating activity of enzymes and transcription factors, and interacting with other signalling molecules like phytohormones (e.g., ABA, SA, and JA) (Kumar & Ohri 2023). To investigate the action of NO in plants, the widely used NO donor, sodium nitroprusside (SNP), is commonly employed. Being a source of NO, SNP is often used to change production of secondary metabolites in plants (Li *et al*. 2011; Ge *et al*. 2019; Farouk & Al‐Huqail 2020). Applying 200 mM SNP resulted in a significant increase in metabolite production in both leaves and roots 2 and 4 days after treatment (Fig. 5b). In a recent study, SNP alone and in combination with SA enhanced activity of enzymatic and non‐enzymatic antioxidants to suppress salinity stress‐induced oxidative damage (Hajihashemi *et al*. 2022). This suggests that priming with SA and SNP can effectively combat salt stress by improving the redox status of plants. In the present study, SNP was one of the most effective treatments for promoting accumulation of major metabolites in *E. velutina* leaves and roots. This finding suggests that NO has a pivotal role in modulating alkaloid and flavonoid profiles in the stress‐related redox challenging conditions of the Caatinga. This capacity also indicates potential use of SNP both as a stand‐alone strategy and in combination with other elicitors to enhance *E. velutina* production of bioactive defence‐ and stress‐related metabolites. This combined approach would mainly involve applications of MeJA and SA, which together with SNP were the phytohormones that yielded higher overall accumulation of compounds in the present study (Fig. 5b).

In the SNP experiments, a peak at the beginning of the chromatographic run (RT <5 min), with the highest absorbance among all treatments, was observed only in treated samples (Fig. 4f). Interestingly, Fraisse *et al*. (2018) analyzed an aqueous extract of *Aloysia triphylla* leaves spiked with SNP and found a major peak at the start of the chromatographic run, detected by HPLC at 280 nm, but this peak was not identified. It is possible that this unidentified molecule with presumably high polarity could be SNP itself or a related derivative. Further research is needed, including the use of analytical tools for molecular identification, to confirm the identity of this molecule.

In our previous research, several alkaloids and flavonoids were identified in *E. velutina*, being important phytochemical marker candidates for this genus (Chacon *et al*. 2021, 2022). However, distinguishing between these two classes of compounds can be difficult as their spectra in the UV region are similar. The aim of the current study was to produce a wide overview of metabolite profiles by analysing hundreds of samples in a comparative manner. To differentiate between these two classes of compounds, HPLC‐DAD patterns of known chemical entities representative of these metabolite classes were obtained (Fig. S1). Examination of chromatograms of samples treated with environmental stressors or with phytohormones showed that most of the accumulated peaks eluted in the intermediate portion of the chromatogram (15 min < RT < 20 min). This suggests that alkaloids are the main class of compounds induced by the different stress factors. However, in some specific treatments, such as temperature, mechanical damage, SA, and water limitation, there was also an accumulation of metabolites in the final portion of the chromatographic run, which is indicative of flavonoids. This profile was particularly recorded in roots.

Stress sensing in roots can lead to overall concerted adjustment with shoots, or impaired plant function. As part of the adaptive response, root antioxidant defences, such as production of flavonoids, may be triggered to counteract the harmful effects of stressful conditions (Winkel‐Shirley 2002; Genzel *et al*. 2021). In addition, the signalling role of several flavonoids exuded by roots for attraction of both nodule‐forming N‐fixing bacteria and mycorrhizal fungi may be important to improve mineral nutrition and water status, thereby supporting overall metabolism (Liu & Murray 2016). Importantly, this metabolic support also includes alkaloid biosynthesis, which relies significantly on N availability and is often found in Fabaceae that associate with N‐fixing bacteria (Wink 2013).

Plant natural products can generally be classified based on their accumulation profiles as phytoanticipins or phytoalexins, which are compounds present in plants either constitutively or induced in response to an elicitor, respectively (VanEtten *et al*. 1994; González‐Lamothe *et al*. 2009; Ejike *et al*. 2013). The chemical fingerprint analysis herein described indicates that the most responsive treatments result in increased accumulation of major metabolites already present in their respective controls, i.e., phytoanticipin‐like metabolites. Nonetheless, phytoalexin‐like metabolites have also been observed. The dominance of phytoanticipin over phytoalexin‐like compounds in *E. velutina* chemical profiles may reflect a likely advantage of this mode of defence in harsh environments, such as the Caatinga, i.e., pre‐accumulation may be metabolically more cost‐effective compared to repeated inducibility over time. As bioactivities and detailed identities of molecules are pursued, the spatiotemporal dynamic chemometric landscape established in the present study sheds light on the adaptive nature of individual specialized metabolites. Moreover, useful guidance for treatment selection and accumulation profile aimed at target molecules of interest from *E. velutina* may also emerge.

Based on the spatiotemporal chemometric patterns described above, we employed a targeted analysis using commercial or isolated standards of select alkaloids and flavonoids previously isolated by our research group. Retention times from these standards were compared with those from control and treated samples to screen for putative matches and to statistically analyse peak areas. Although the HPLC‐DAD method does not provide full compound identification, it enables comparative inferences based on retention time similarity, supporting its use for tentative identification of flavonoid and alkaloid chromophores. Of the nine standards tested, the alkaloids erysodine/erysovine (isomers), erythraline, and the flavonoid quercetin were selected for comparison. The select alkaloids were chosen because of their known abundance in the *Erythrina* genus, prior isolation, and characterization by our group (de Almeida *et al*. 2021). The stress conditions selected for peak area analysis – high temperature (50°C), methyl jasmonate (40 μM), sodium nitroprusside (200 mM), and prolonged water deficit (3 weeks) – were those that markedly increased early to intermediate retention time peaks (RT < 20 min), corresponding to the alkaloid range (Fig. S1). Under these conditions, peaks consistent with erysodine/erysovine showed a significant increase across all treatments (*P* < 0.05; Fig. S26a), whereas erythraline‐like peaks exhibited only a slight, non‐significant elevation under high temperature (Fig. S26b).

Among the flavonoids, quercetin exhibited one of the highest retention time matches with peaks in the experimental chromatograms. In a previous study, we also identified quercetin‐analogous compounds and observed modulation of their associated gene expression (Chacon *et al*. 2022). Peaks consistent with quercetin were predominantly detected in roots after 2 days of exposure to various stressors, including sodium nitroprusside (SNP), mechanical damage, salicylic acid (SA), NaCl, and UV light. Among these, SA (1 mM) significantly increased quercetin‐like peaks (*P* < 0.05; Fig. S26c), while SNP and NaCl treatments showed substantial size effects, suggesting potential biological relevance despite the absence of statistical significance (Fig. S26d).

Although this approach does not enable full metabolite identification, it supports the hypothesis that specific stressors selectively modulate the biosynthesis of alkaloid and flavonoid classes. This targeted screening phase is essential for identifying stressors that elicit pronounced chemical responses, thus guiding future high‐resolution metabolomic analyses aimed at fully annotating bioactive compounds.

## CONCLUSIONS AND PERSPECTIVES

Profiles of specialized metabolites and plant physiological status have a complex relationship. The spatial–temporal intertwined regulatory routes involved require complementary experimental approaches to increase understanding. Herein, the metabolomic strategy of metabolic fingerprinting provided useful insights for *E. velutina*, an under‐studied species of medicinal value originating in a unique semiarid biome. Clearly, several signals can modulate tree yield and profile of natural products, thereby enhancing its adaptive and, perhaps, pharmaceutical potential. Key moderate stress treatments (applied as such or mimicked using phytohormones) impacting phytoalexin and phytoanticipin production were mapped. However, to further validate these findings, studies with combinations of multiple stress factors simultaneously and/or sequentially should be examined. Moreover, most relevant treatments for the accumulation of metabolites should be coupled to additional analytical techniques, such as mass spectrometry, to ascertain the exact identity of individual molecules. Additional experiments addressing the molecular identity of the metabolites as well as evaluations of expression data on the major candidate genes in the biosynthetic pathways of alkaloids and flavonoids of *E. velutina* are ongoing. This important and resilient natural and medicinal plant resource, although far from being a model species, can contribute to building a detailed and dynamic picture of the Caatinga's unique phytochemical richness and molecular identity.

## AUTHOR CONTRIBUTIONS

CTC, JV, and RBG carried out the cultivation, germination, and application of elicitor treatments. DSC and MK performed extraction of metabolites and analysis in liquid chromatography. JASZ provided the HPLC‐DAD resources. DSC, BB, and LP performed univariate and multivariate statistical analysis of chemical data. DSC, BB, LP, CTC, JV, and JASZ participated in discussions of the entire study. AFN and RBG coordinated the entire study, participated in the discussions, and finalized the manuscript. All authors discussed the main conclusions and contributed to writing of the manuscript.

## FUNDING

This work was supported by Instituto Serrapilheira (grant no. Serra‐1709‐19691), the National Institute of Science and Technology in Biodiversity and Natural Products (INCT‐BioNat) [grant no. 465637/2014–0], the National Council for Scientific and Technological Development (CNPq) (grant no. 310775/2021–3), and the Coordination for the Improvement of Higher Education Personnel ‐ Brazil [(CAPES) ‐ Finance Code 001]. L.P acknowledges the Fundação de Amparo à Pesquisa no Rio de Janeiro (FAPERJ) (grant no. E‐26/201.928/2020), and Universidade do Estado do Rio de Janeiro (Programa Pró‐Ciência) for financial support.

## CONFLICT OF INTEREST

The authors declare no conflicts of interest.

## Supporting information


**Data S1.** Overview of statistical analyses and data sets of responses to environmental and phytohormone‐related stress factors.
**Fig. S1.** Chromatogram with elution peaks of the analyzed external standards.
**Fig. S2.** HPLC fingerprints for heat treatment.
**Fig. S3.** Plots of scores (left) and loadings (right) of ANOVA ‐PCA results for temperature treatment.
**Fig. S4.** HPLC fingerprints for UV light treatment.
**Fig. S5.** Plots of scores (left) and loadings (right) of ANOVA ‐PCA results for UV ‐C treatment.
**Fig. S6.** HPLC fingerprints for water restriction treatment in leaves.
**Fig. S7.** HPLC fingerprints for water restriction treatment in roots.
**Fig. S8.** Plots of scores (left) and loads (right) of ANOVA ‐PCA results for drought treatment.
**Fig. S9.** HPLC fingerprints for saline stress treatment.
**Fig. S10.** Plots of scores (left) and loads (right) of ANOVA‐PCA results for saline stress treatment.
**Fig. S11.** HPLC fingerprints for mechanical damage treatment.
**Fig. S12.** Plots of scores (left) and loads (right) of ANOVA‐PCA results for mechanical damage treatment.
**Fig. S13.** HPLC fingerprints for methyl jasmonate (MeJA) treatment.
**Fig. S14.** Plots of scores (left) and loads (right) of ANOVA‐PCA results for methyl jasmonate (MeJA) treatment.
**Fig. S15.** HPLC fingerprints for salicylic acid (SA) treatment.
**Fig. S16.** Plots of scores (left) and loads (right) of ANOVA ‐PCA results for salicylic acid (SA) treatment.
**Fig. S17.** HPLC fingerprints for nitric oxide (NO) treatment supplied as sodium nitroprusside (SNP).
**Fig. S18.** Plots of scores (left) and loads (right) of ANOVA‐PCA results for nitric oxide (NO) treatment supplied as sodium nitroprusside (SNP) treatment.
**Fig. S19.** HPLC fingerprints for abscisic acid (ABA) treatment.
**Fig. S20.** Plots of scores (left) and loads (right) of ANOVA‐PCA results for abscisic acid (ABA) treatment.
**Fig. S21.** Overlay graphs of water restriction after 3 weeks (T3), temperature (50°C), and UV light (48h) chromatograms.
**Fig. S22.** Overlay graphs of water limitation after one, two and three weeks.
**Fig. S23.** Overlay graphs of water limitation after three weeks (T3) and ABA 95 μM and 189 μM.
**Fig. S24.** Overlay graphs of MeJA (40 μM) and mechanical damage (75% of leaf area removal).
**Fig. S25.** Overlay graphs of MeJA (40 μM) and Salicylic acid (1 mM).
**Fig. S26.** Bar plots showing the chromatographic peak areas of compounds classified as (A) Erysodine/Erysovine‐like, (B) Erythraline‐like, and (C, D) Quercetin‐like under different experimental conditions.
**Scheme S1.** Developmental series of seed germination and growth of *Erythrina velutina*.
**Table S1.** Experimental design of treatments for *Erythrina velutina* leaves and roots.
**Table S2.** Mobile phase gradient used for experiments in HPLC‐DAD.
**Table S3.** Patterns of alkaloids and flavonoids injected into HPLC‐DAD. RT: retention time in minutes.
**Table S4.** Treatment table and evaluation of alignment/non‐alignment.

## Data Availability

Data sharing is not applicable to this article; however, data can be made accessible upon a reasonable request to the corresponding author. The statistical results can be accessed at the following link: https://docs.google.com/spreadsheets/d/14zOewfcKSWonq9oz8HfA3Crmd80KUChu/edit?usp=sharing&ouid=104723688721725468234&rtpof=true&sd=true.
