# Supplementary material for: Environmental and phytohormone modulation of organ‐specific specialized metabolite profiles in the dryland tree Erythrina velutina
Source: Plant Biol (Stuttg). 2025 Sep 8;27(7):1326–40. doi: 10.1111/plb.70104 (PMC12631523; doi:10.1111/plb.70104)
Supplement: Supplementary file 1 — Data S1. Overview of statistical analyses and data sets of responses to environmental and phytohormone‐related stress factors. Fig. S1. Chromatogram with elution peaks of the analyzed external standards. Fig. S2. HPLC fingerprints for heat treatment. Fig. S3. Plots of scores (left) and loadings (right) of ANOVA ‐PCA results for temperature treatment. Fig. S4. HPLC fingerprints for UV light treatment. Fig. S5. Plots of scores (left) and loadings (right) of ANOVA ‐PCA results for UV ‐C treatment. Fig. S6. HPLC fingerprints for water restriction treatment in leaves. Fig. S7. HPLC fingerprints for water restriction treatment in roots. Fig. S8. Plots of scores (left) and loads (right) of ANOVA ‐PCA results for drought treatment. Fig. S9. HPLC fingerprints for saline stress treatment. Fig. S10. Plots of scores (left) and loads (right) of ANOVA‐PCA results for saline stress treatment. Fig. S11. HPLC fingerprints for mechanical damage treatment. Fig. S12. Plots of scores (left) and loads (right) of ANOVA‐PCA results for mechanical damage treatment. Fig. S13. HPLC fingerprints for methyl jasmonate (MeJA) treatment. Fig. S14. Plots of scores (left) and loads (right) of ANOVA‐PCA results for methyl jasmonate (MeJA) treatment. Fig. S15. HPLC fingerprints for salicylic acid (SA) treatment. Fig. S16. Plots of scores (left) and loads (right) of ANOVA ‐PCA results for salicylic acid (SA) treatment. Fig. S17. HPLC fingerprints for nitric oxide (NO) treatment supplied as sodium nitroprusside (SNP). Fig. S18. Plots of scores (left) and loads (right) of ANOVA‐PCA results for nitric oxide (NO) treatment supplied as sodium nitroprusside (SNP) treatment. Fig. S19. HPLC fingerprints for abscisic acid (ABA) treatment. Fig. S20. Plots of scores (left) and loads (right) of ANOVA‐PCA results for abscisic acid (ABA) treatment. Fig. S21. Overlay graphs of water restriction after 3 weeks (T3), temperature (50°C), and UV light (48h) chromatograms. Fig. S22. Overlay graphs of water limitation after [file PLB-27-1326-s001.docx]

**Environmental and phytohormonal modulation of organ-specific specialized metabolite profiles in the dryland tree *Erythrina velutina***

**Daisy Sotero Chacon^a#^, Bernardo Bonilauri^b#^, Cibele Tesser da Costa^c^, Johnatan Vilasboa^c^, Mariana Koetz^e^, Licarion Pinto^d^, José Angelo Silveira Zuanazzi^e^, Raquel Brandt Giordani^a*^, Arthur Germano Fett-Neto^c*^**

^a^ Department of Pharmacy; Federal University of Rio Grande do Norte (UFRN); Natal, RN, Brazil

^b^ Stanford Cardiovascular Institute, Stanford University School of Medicine, Stanford, CA, USA

^c^ Plant Physiology Laboratory, Center for Biotechnology and Department of Botany, Federal University of Rio Grande do Sul, Porto Alegre, RS, Brazil

^d^ Department of Analytical Chemistry (DQA), State University of Rio de Janeiro (UERJ), Rio de Janeiro, RJ, Brazil

^e^ Laboratory of Pharmacognosy, Federal University of Rio Grande do Sul, Porto Alegre, RS, Brazil

#Daisy Sotero Chacon and Bernardo Bonilauri contributed equally to this work

***Correspondence:**Raquel Brandt Giordani ([raquel.giordani@](rewritten://8e66e545-e1b3-43eb-a219-196357f40620)ufrn.br, telephone number: +55(84) 3342-9818) and Arthur G. Fett-Neto
([fett.neto@ufrgs.br](mailto:fett.neto@ufrgs.br), telephone number: +55(51) 3308-7642)


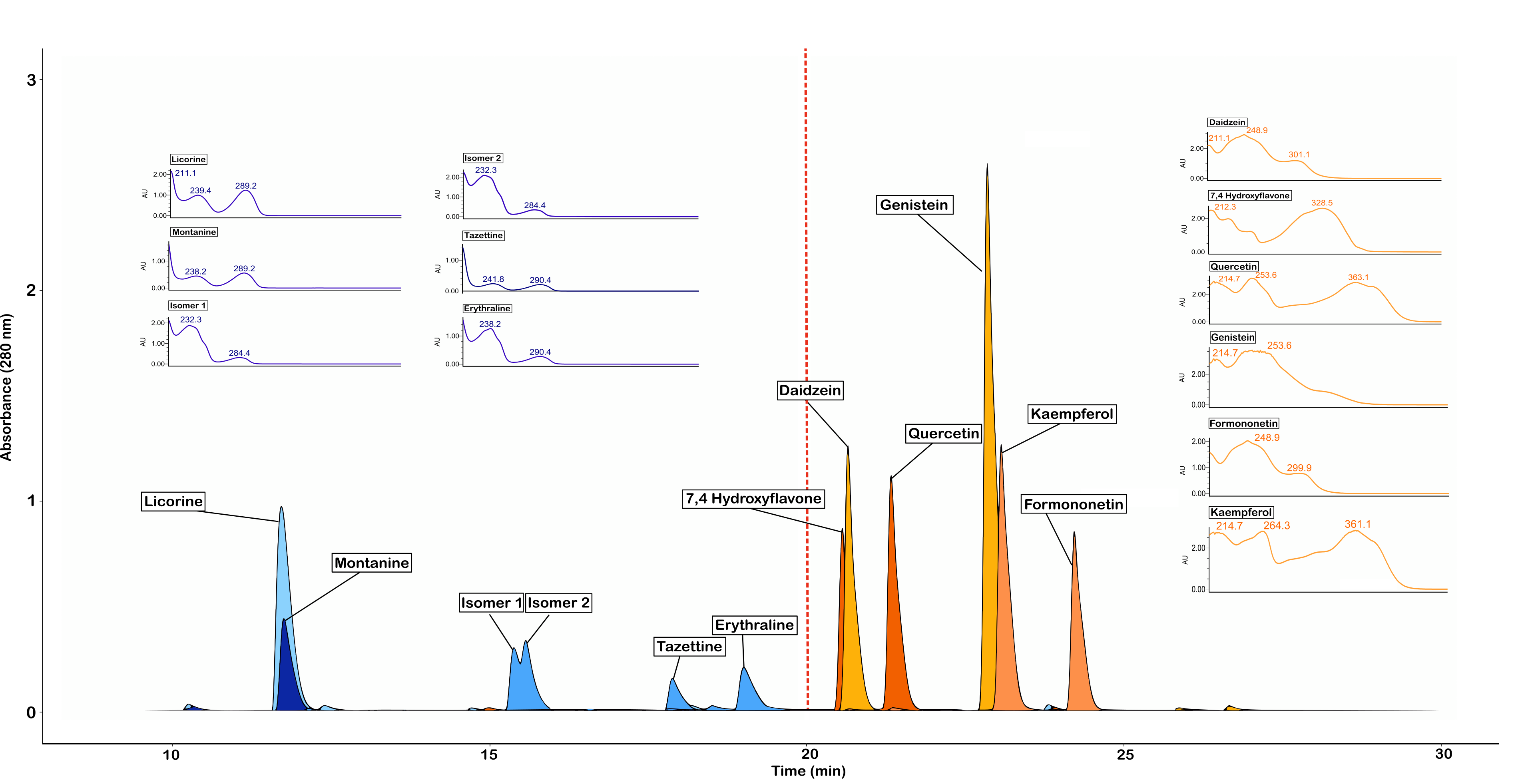


**Figure S1. Chromatogram with elution peaks of the analyzed external standards.** X and Y axes represent the retention time during the chromatographic run and the number of absorbance units, respectively. Orange and blue peaks represent metabolites of the flavonoid and alkaloid classes, respectively.


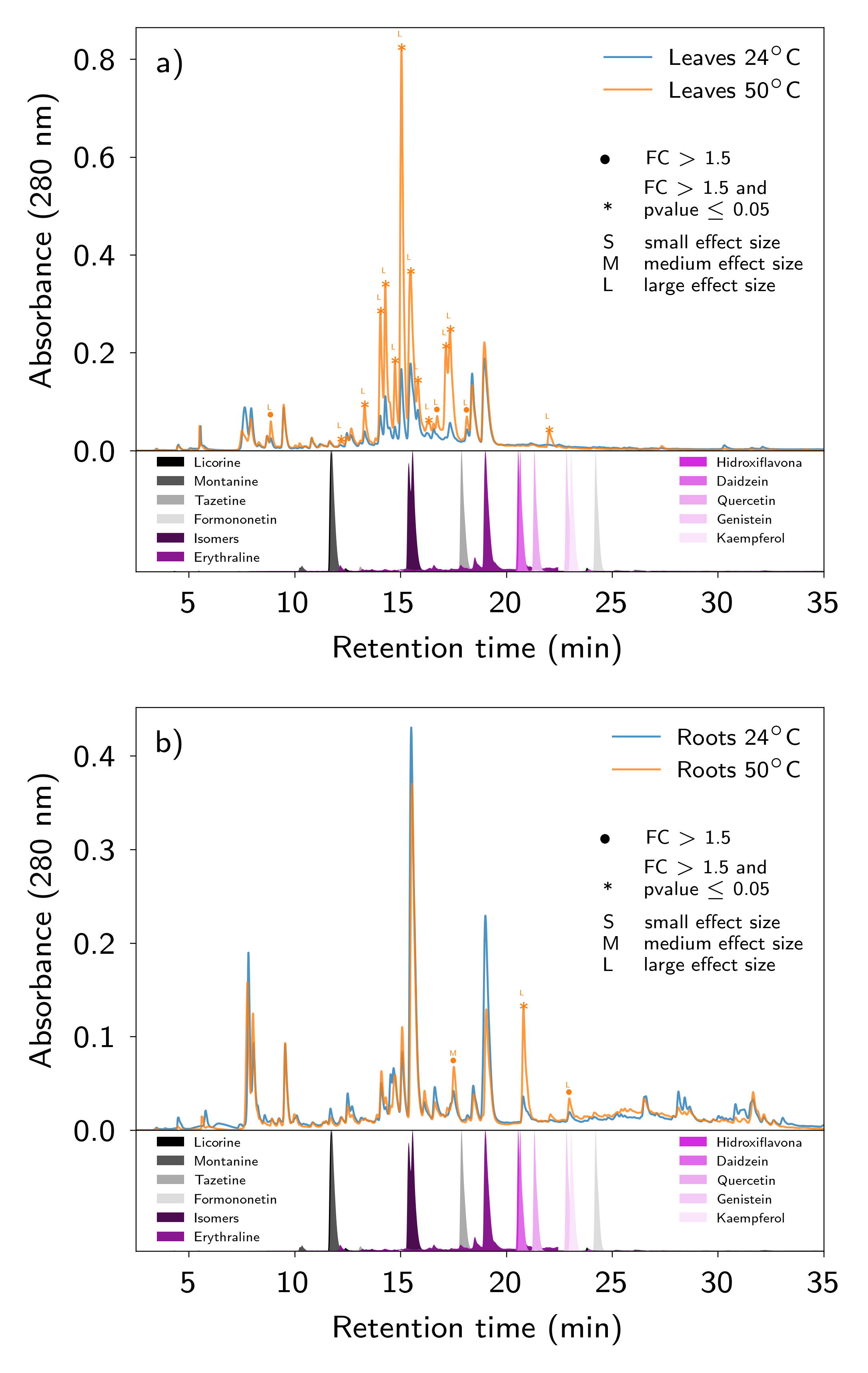


**Figure S2. HPLC fingerprints for heat treatment.** The blue chromatograms correspond to control samples maintained at 24°C, while the orange chromatograms depict the results of samples subjected to 50°C treatment for both leaves (a) and roots (b). To improve the interpretation of the data resulting from heat treatment, the average of replicates was taken to obtain a single value for each time point, and a cutoff was applied at retention time of 35 minutes to focus on the main peaks. •Colored circles indicate peaks with a fold change (FC) greater than 1.5, with each color representing a corresponding experimental group. *Colored asterisks indicate peaks with FC > 1.5 and p-value < 0.05, with each color representing a corresponding experimental group. The colored peaks in the lower part of the chromatogram represent the injected standard of alkaloids and flavonoids, with purple indicating compounds previously identified in *Erythrina* genus. Each group consisted of n = 5 biological replicates for leaves and roots (for details see Table S1).


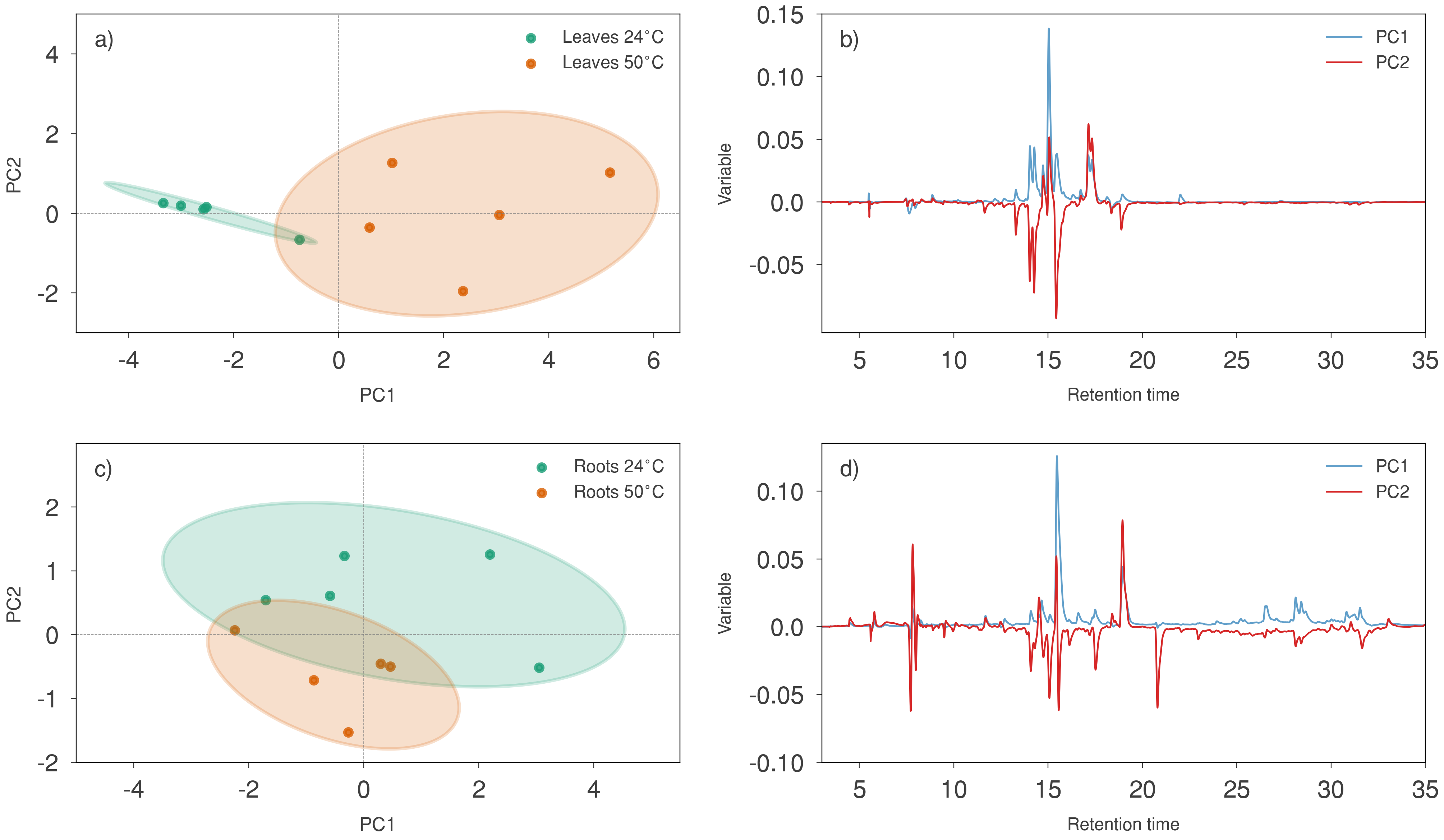


**Figure S3. Plots of scores (left) and loadings (right) of ANOVA -PCA results for temperature treatment.** Temperature treatment analysis was performed for leaves (a and b) and roots (c and d) for 24°C vs. 50°C. The principal components are highlighted with different colors. Ellipse regions in the score graphs represent the region of confidence by class (95%). A threshold was applied at 35 minutes retention time to focus on the main peaks.


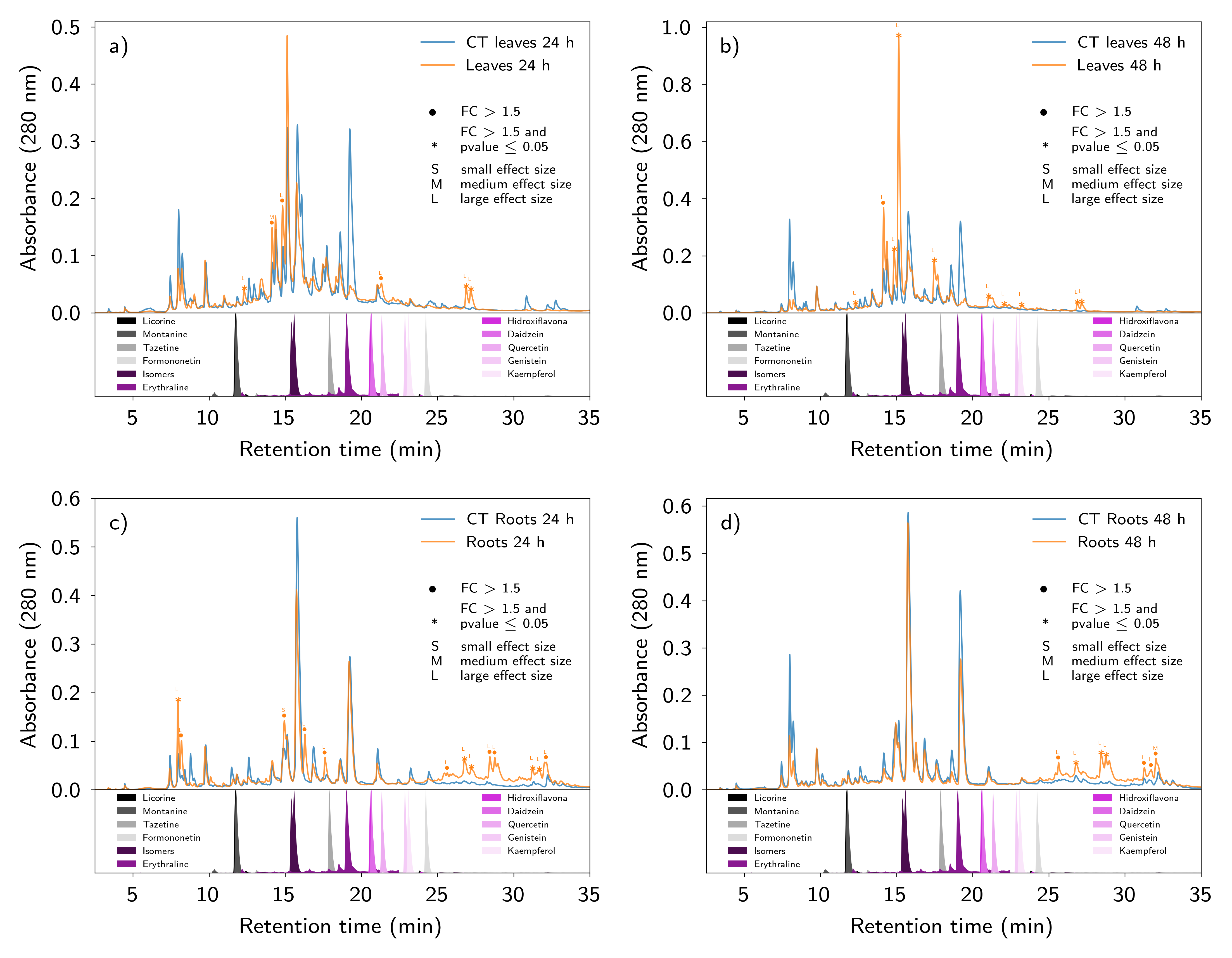


**Figure S4. HPLC fingerprints for UV light treatment.** The orange chromatograms represent the results of samples exposed to ultraviolet light in 24h leaves (a), 48h leaves (b), 24h roots (c), and 48h roots (d). The blue chromatograms correspond to control samples of their respective treatments. The chromatographic data from replicates were averaged to obtain a single value for each time point, and a threshold was applied at 35 minutes retention time to focus on the main peaks. •Colored circles indicate peaks with a fold change (FC) greater than 1.5, with each color representing a corresponding experimental group. *Colored asterisks indicate peaks with FC > 1.5 and p-value < 0.05, with each color representing a corresponding experimental group. The colored peaks in the lower part of the chromatogram represent the injected standard of alkaloids and flavonoids, with purple indicating compounds previously identified in *Erythrina* genus. Each group consisted of n = 5 biological replicates for leaves and *n*=4 or 5 biological replicates for roots (for details see Table S1).


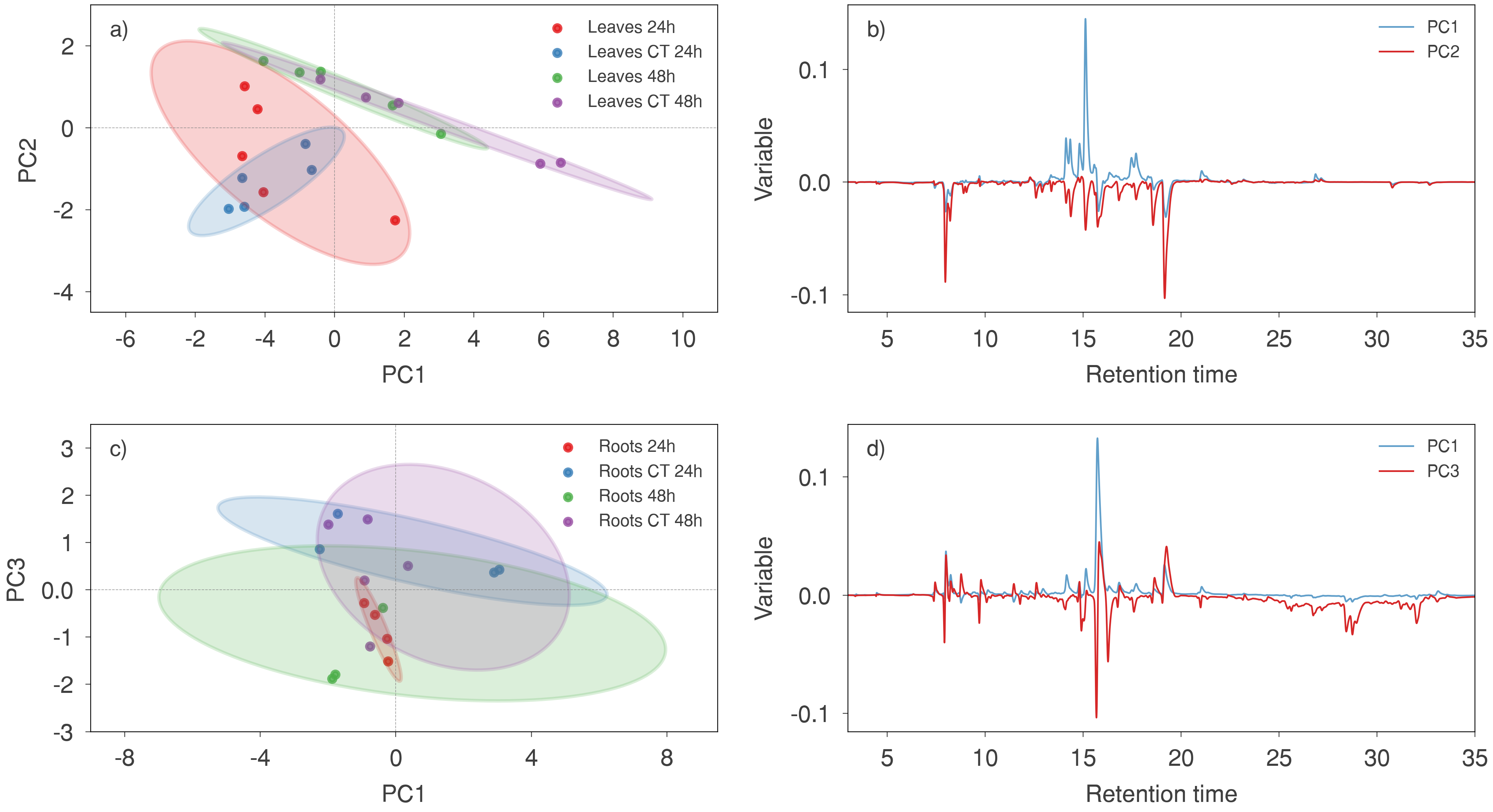


**Figure S5. Plots of scores (left) and loadings (right) of ANOVA -PCA results for UV -C treatment.** UV-C treatment analysis was performed for leaves (a and b) and roots (c and d) for 24h and 48h. The principal components are highlighted with different colors. Ellipse regions in the score graphs represent the region of confidence by class (95%). A threshold was applied at 35 minutes retention time to focus on the main peaks.


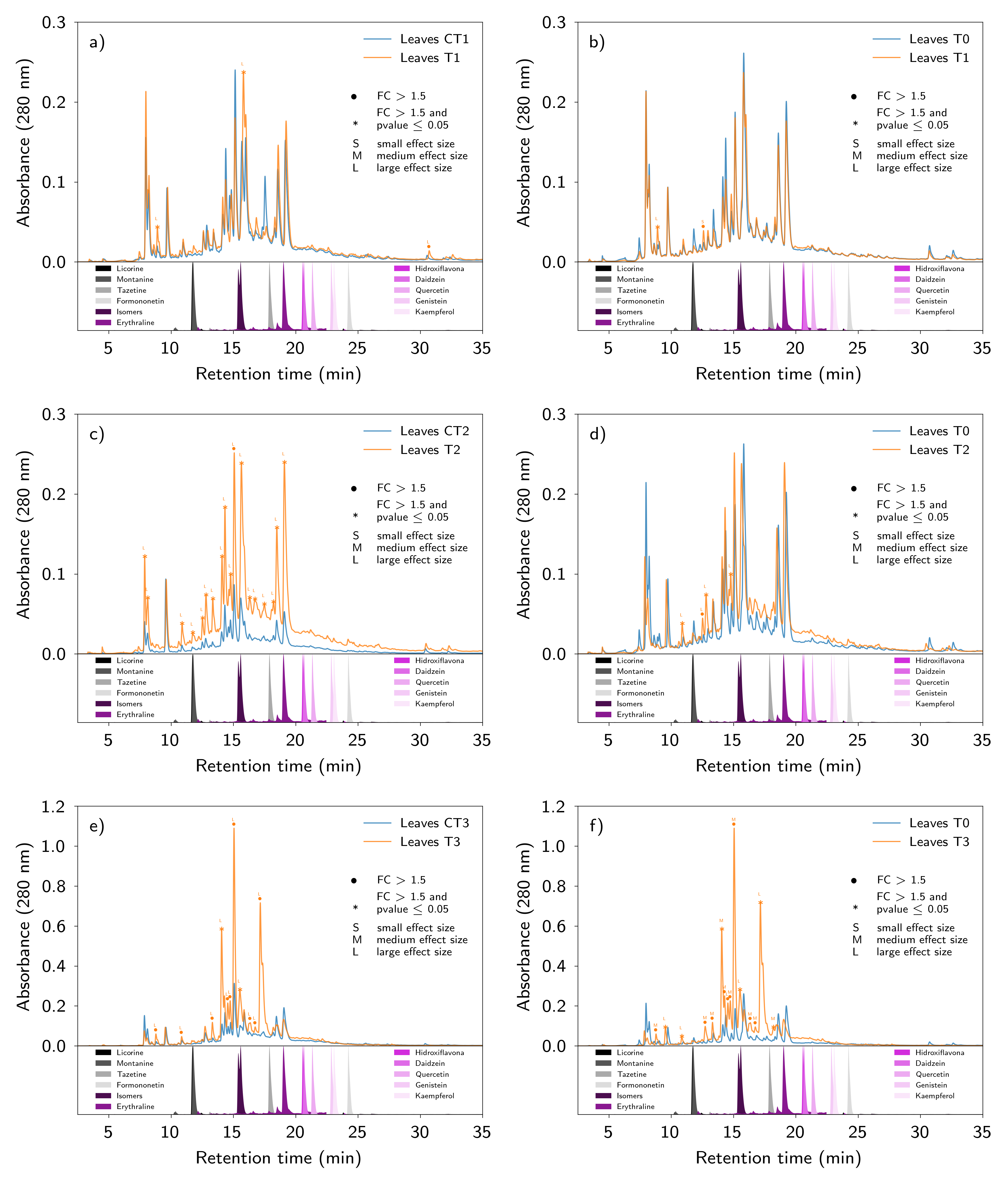
**Figure S6. HPLC fingerprints for water restriction treatment in leaves.** Orange chromatograms represent the chemical profiles of the samples in one (T1), two (T2) and three (T3) weeks of irrigation suspension. (a), (c) and (e) represent samples T1, T2 and T3 compared to their respective irrigated control (shown in blue) during one, two and three weeks; (b), (d) and (f) represent samples T1, T2 and T3 compared to the irrigated control at the start of experiment (T0) (shown in blue). The replicates of chromatographic data were averaged to obtain a single value for each time point, and a limit was applied at 35 minutes retention time to focus on the main peaks. •Colored circles indicate peaks with a fold change (FC) greater than 1.5, with each color representing a corresponding experimental group. *Colored asterisks indicate peaks with FC > 1.5 and p-value < 0.05, with each color representing a corresponding experimental group. The colored peaks in the lower part of the chromatogram represent the injected standard of alkaloids and flavonoids, with purple indicating compounds previously identified in *Erythrina* genus. Each group consisted of n = 5 biological replicates for leaves (for details see Table S1).


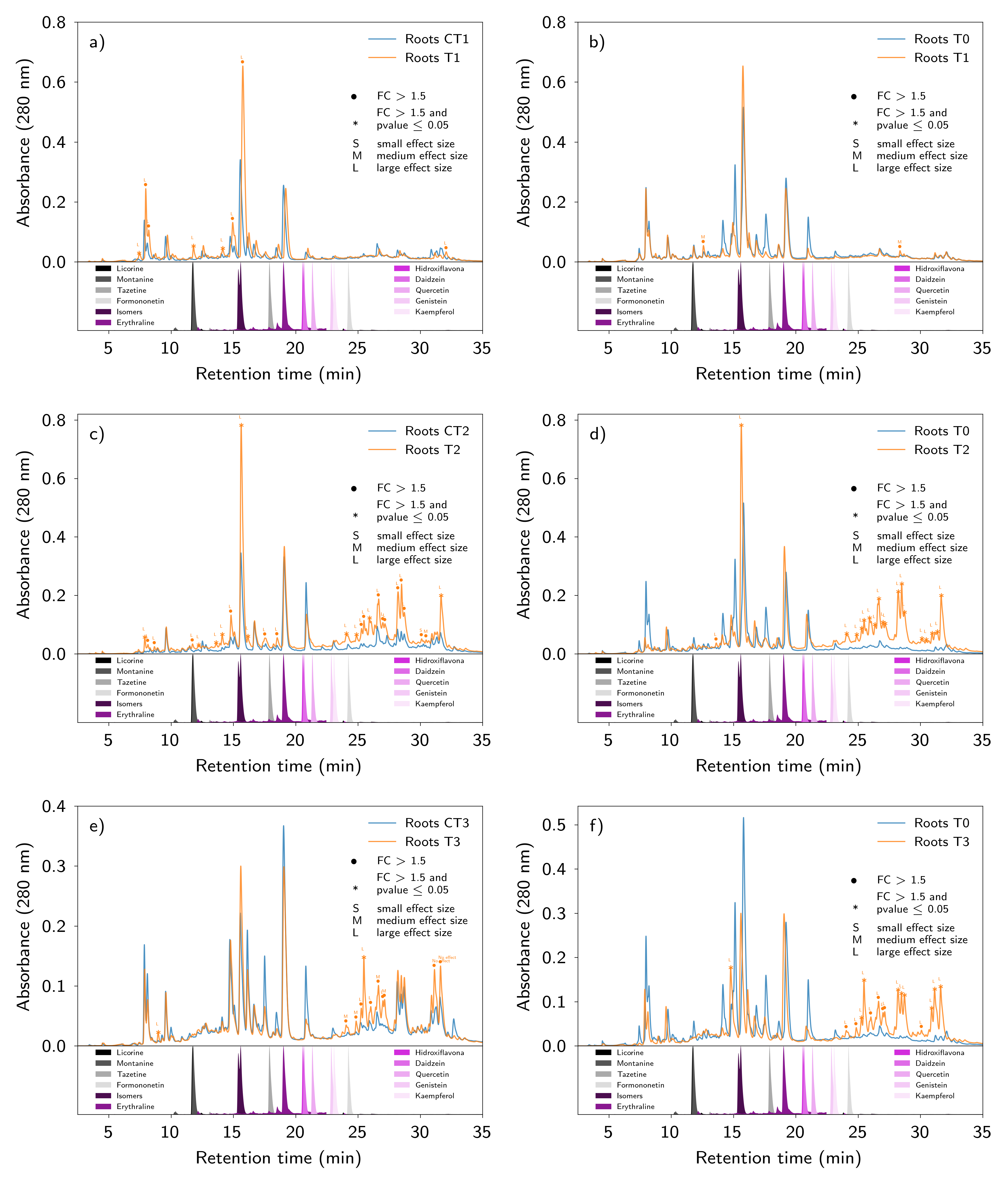
**Figure S7. HPLC fingerprints for water restriction treatment in roots.** Orange chromatograms represent the chemical profiles of the samples in one (T1), two (T2) and three (T3) weeks of irrigation suspension. (a), (c) and (e) represent samples T1, T2 and T3 compared to their respective irrigated control (shown in blue) during one, two and three weeks; (b), (d) and (f) represent samples T1, T2 and T3 compared to the irrigated control at the start of experiment (T0) (shown in blue). The replicates of chromatographic data were averaged to obtain a single value for each time point, and a limit was applied at 35 minutes retention time to focus on the main peaks. •Colored circles indicate peaks with a fold change (FC) greater than 1.5, with each color representing a corresponding experimental group. *Colored asterisks indicate peaks with FC > 1.5 and p-value < 0.05, with each color representing a corresponding experimental group. The colored peaks in the lower part of the chromatogram represent the injected standard of alkaloids and flavonoids, with purple indicating compounds previously identified in *Erythrina* genus. Each group consisted of *n*=3 to 5 biological replicates for roots (for details see Table S1).


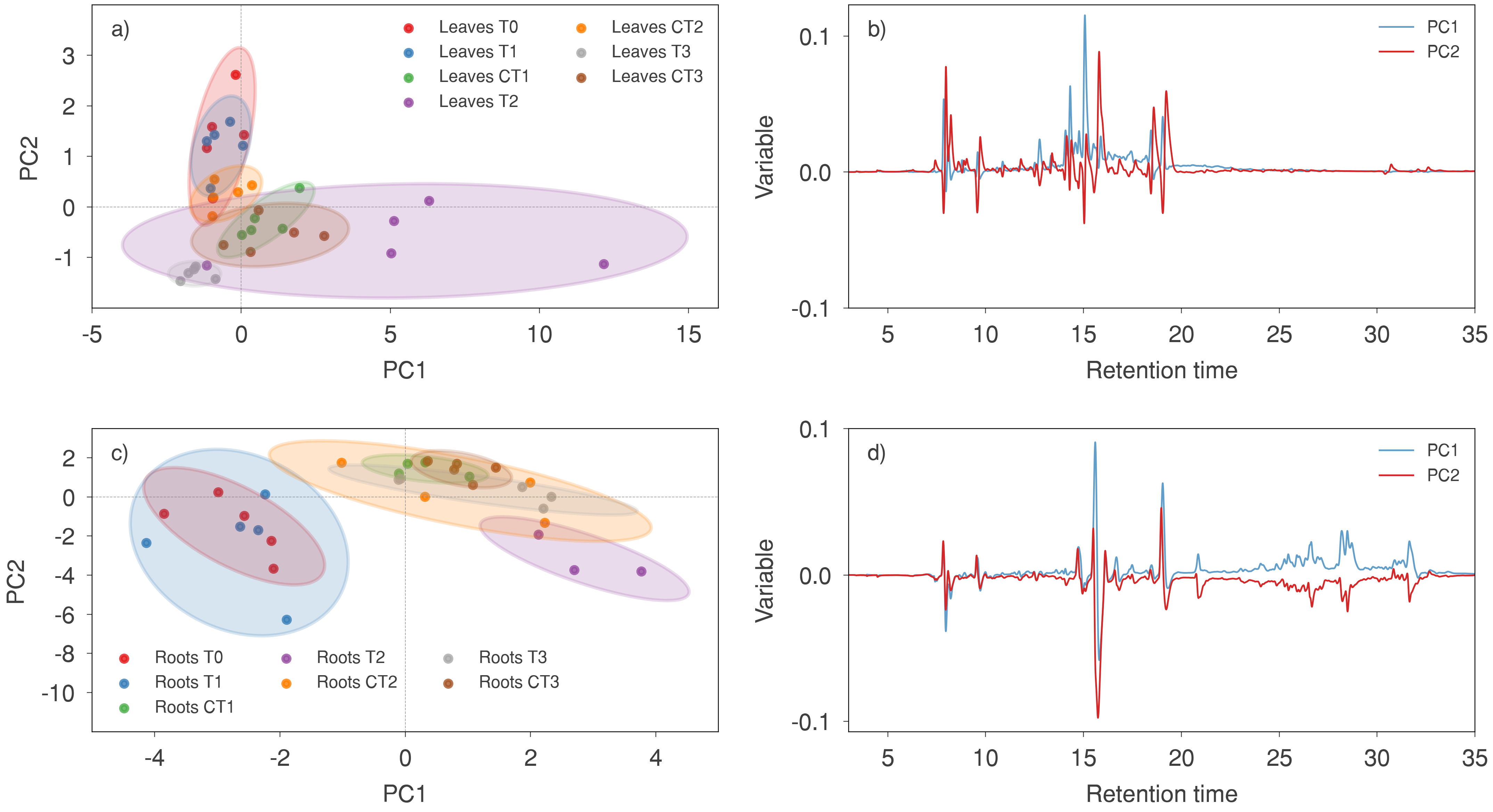


**Figure S8. Plots of scores (left) and loads (right) of ANOVA -PCA results for drought treatment.** Drought treatment analysis was performed for leaves (a and b) and roots (c and d) for one, two and three weeks. The principal components are highlighted with different colors. Ellipse regions in the score graphs represent the region of confidence by class (95%). A threshold was applied at 35 minutes retention time to focus on the main peaks.


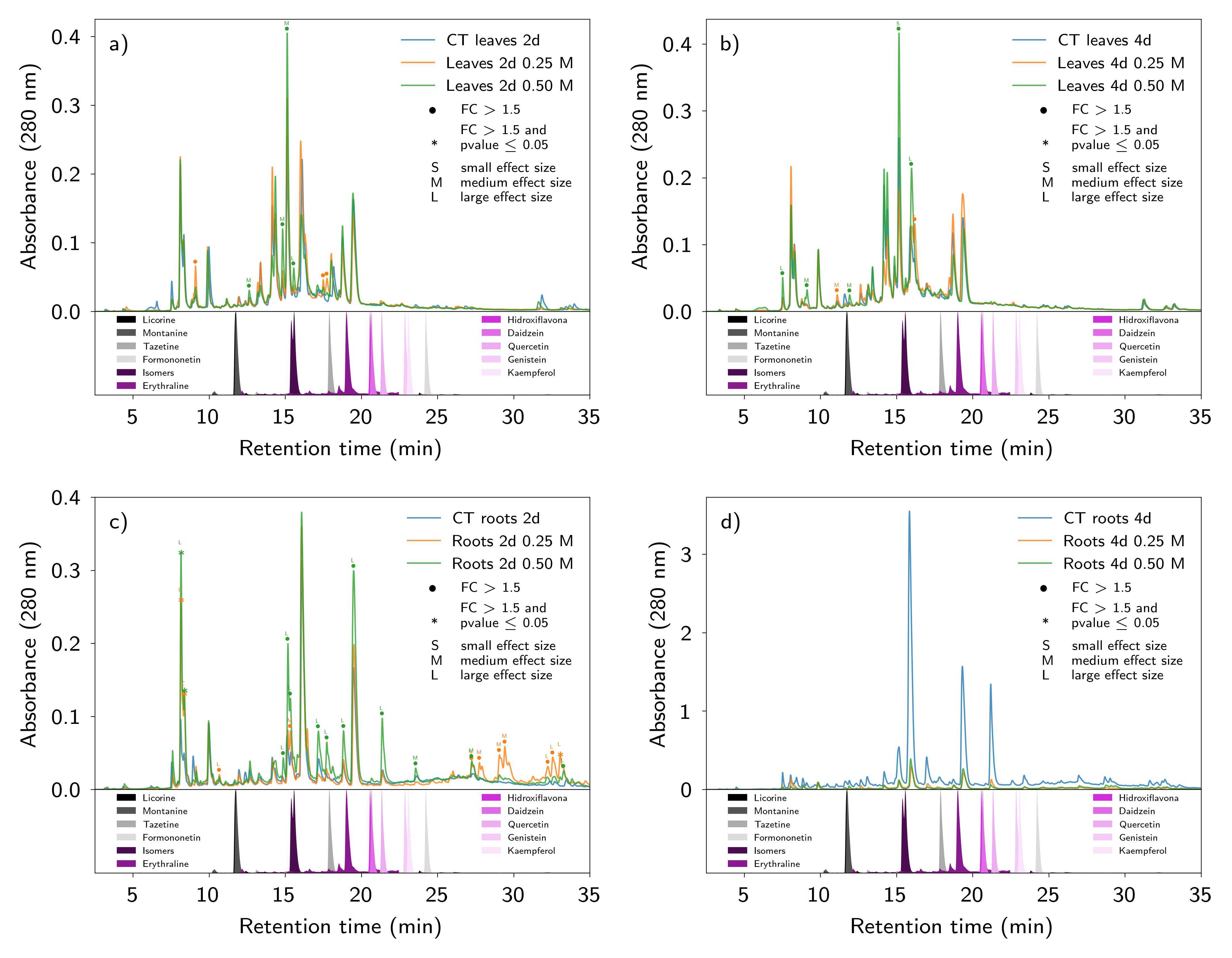


**Figure S9. HPLC fingerprints for saline stress treatment.** Orange chromatograms represent the chemical profiles of samples treated with 0.25 M of the NaCl; green chromatograms represent the chemical profile of treatment with 0.50 M of the NaCl; blue chromatograms represent controls (devoid of added NaCl). (a) and (b) show the chromatograms of leaves at 2 and 4 days, respectively, while (c) and (d) represent the chromatograms of roots at 2 and 4 days, respectively. Chromatographic data from replicates were averaged to obtain a single value for each time point, and a threshold was applied at 35 minutes retention time to focus on major peaks. •Colored circles indicate peaks with a fold change (FC) greater than 1.5, with each color representing a corresponding experimental group. *Colored asterisks indicate peaks with FC > 1.5 and p-value < 0.05, with each color representing a corresponding experimental group. The colored peaks in the lower part of the chromatogram represent the injected standard of alkaloids and flavonoids, with purple indicating compounds previously identified in *Erythrina* genus. Each group consisted of *n*=5 biological replicates for leaves (except for leaves 2d 0.25 M, with *n*=2) and *n*=3 to 5 biological replicates for roots (for details see Table S1).


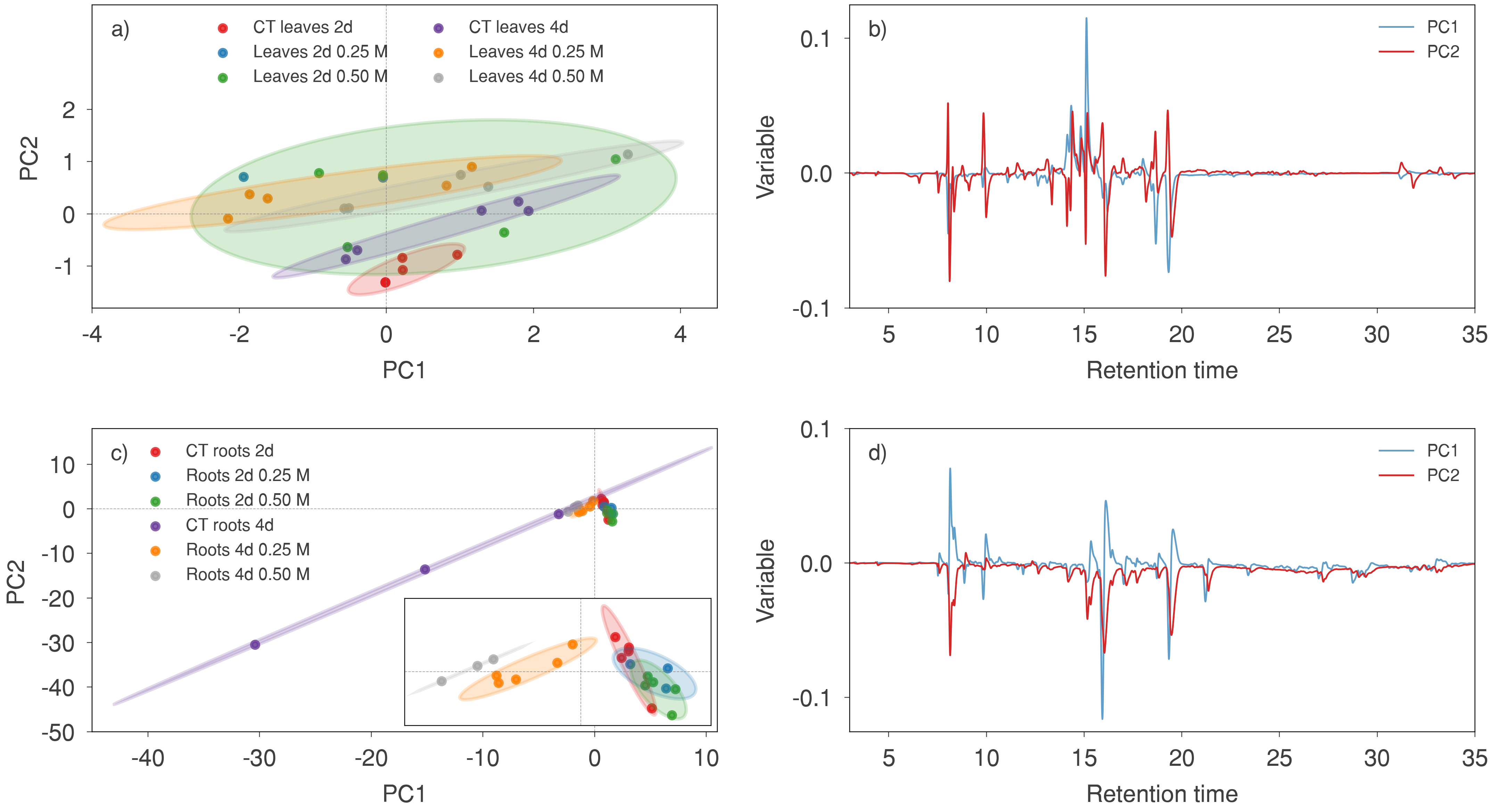


**Figure S10. Plots of scores (left) and loads (right) of ANOVA-PCA results for saline stress treatment.** Saline treatment analysis was performed for leaves (a and b) and roots (c and d), for 2 and 4 days and concentration of 0.25 and 0.50 M. The principal components are highlighted with different colors. Ellipse regions in the score graphs represent the region of confidence by class (95%). A threshold was applied at 35 minutes retention time to focus on the main peaks.


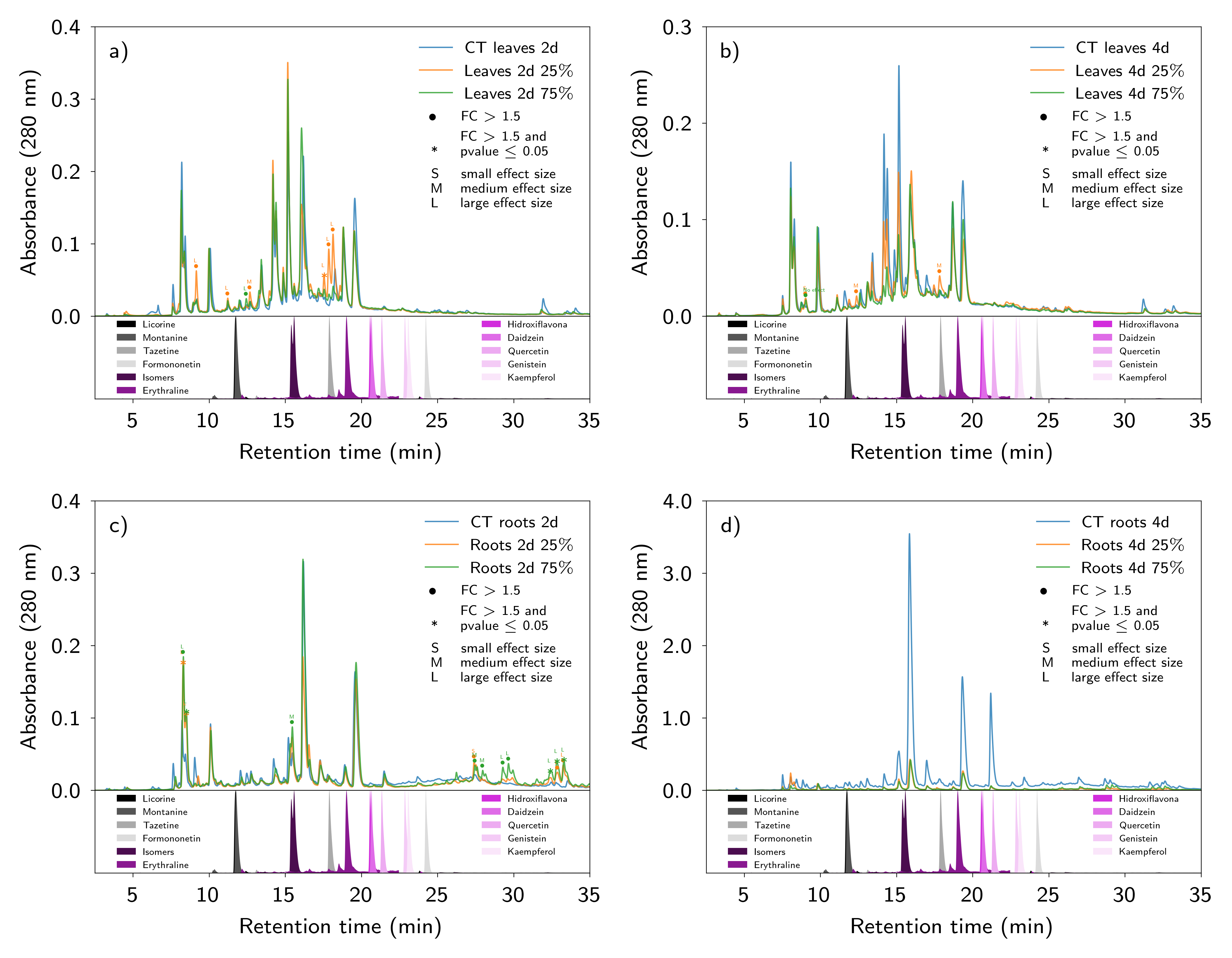


**Figure S11. HPLC fingerprints for mechanical damage treatment.** Orange chromatograms represent the chemical profiles of samples treated with lower mechanical damage intensity (25% of the leaf area); green chromatograms represent the chemical profile upon application of higher mechanical damage intensity (75% of the leaf area); blue chromatograms represent controls (no leaf damage). (a) and (b) show the chromatograms of leaves at 2 and 4 days, respectively, while (c) and (d) represent the chromatograms of roots at 2 and 4 days after mechanical damage, respectively. Chromatographic data from replicates were averaged to obtain a single value for each time point, and a threshold was applied at 35 minutes retention time to focus on major peaks. •Colored circles indicate peaks with a fold change (FC) greater than 1.5, with each color representing a corresponding experimental group. *Colored asterisks indicate peaks with FC > 1.5 and p-value < 0.05, with each color representing a corresponding experimental group. The colored peaks in the lower part of the chromatogram represent the injected standard of alkaloids and flavonoids, with purple indicating compounds previously identified in *Erythrina* genus. Each group consisted of *n*=5 biological replicates for leaves and *n*=3 or 5 biological replicates for roots (for details see Table S1).


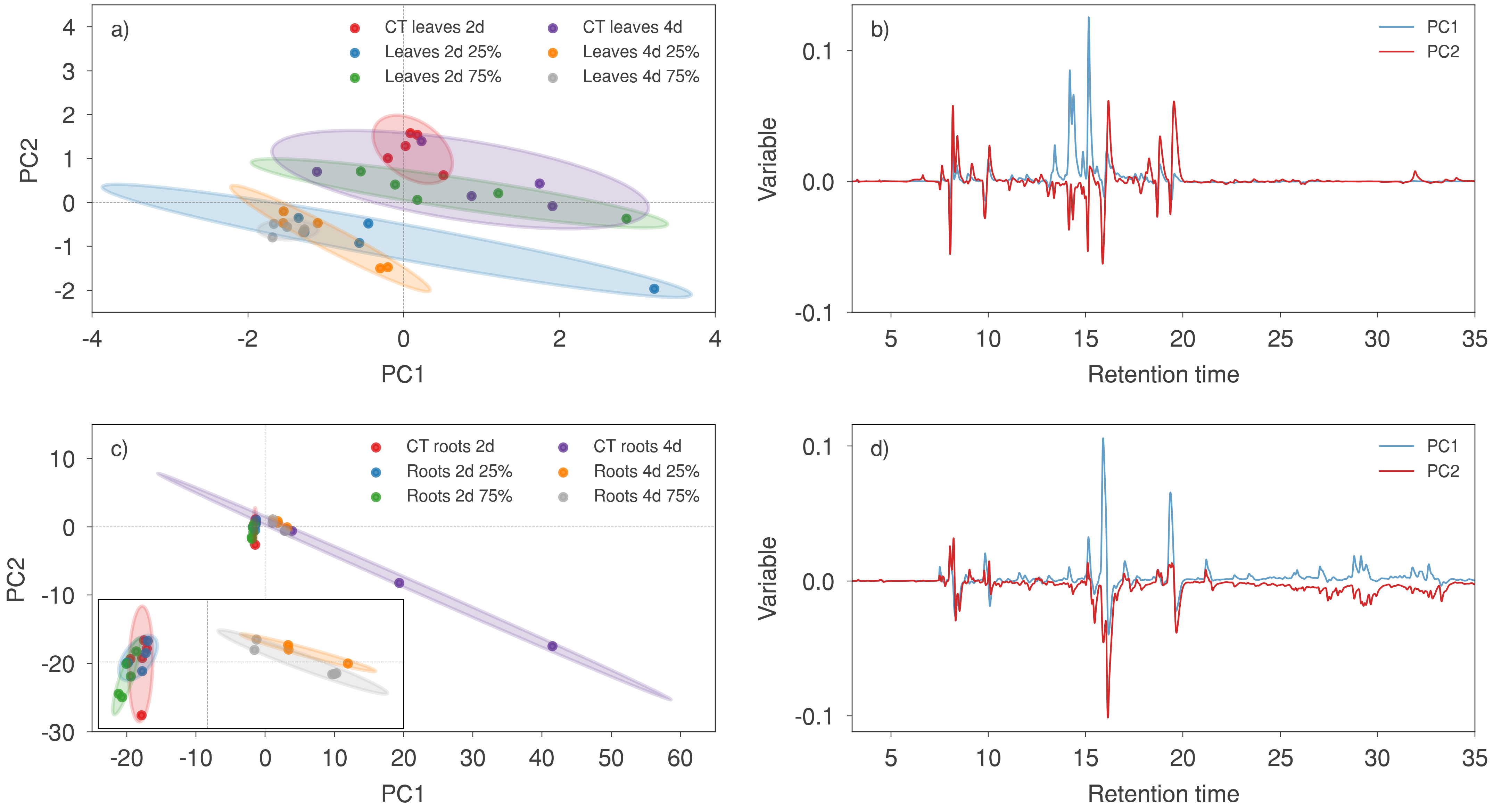


**Figure S12. Plots of scores (left) and loads (right) of ANOVA-PCA results for mechanical damage treatment.** Mechanical damage treatment analysis was performed for leaves (a and b) and roots (c and d), for 2 and 4 days and 25 or 75% of the foliar area. The first and second principal components are highlighted green and orange, respectively. The principal components are highlighted with different colors. Ellipse regions in the score graphs represent the region of confidence by class (95%). A threshold was applied at 35 minutes retention time to focus on the main peaks.


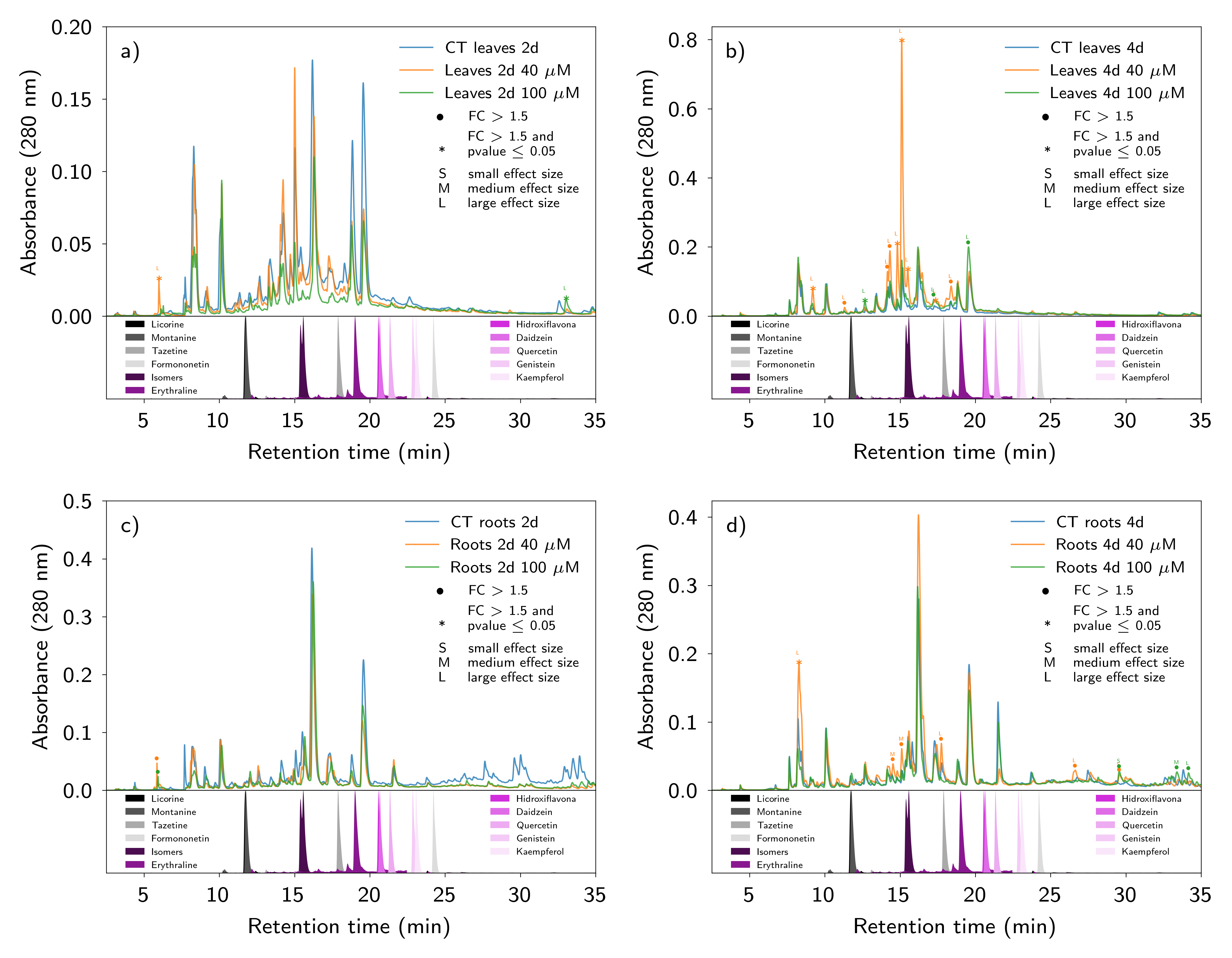


**Figure S13. HPLC fingerprints for methyl jasmonate (MeJA) treatment.** Orange and green chromatograms represent the chemical profiles of samples treated with 40 µM and 100 µM of MeJA, respectively. Blue chromatograms represent controls. (a) and (b) show the chromatograms of leaves at 2 and 4 days, respectively, while (c) and (d) represent the chromatograms of roots at 2 and 4 days after application of MeJA, respectively. Chromatographic data from replicates were averaged to obtain a single value for each time point, and a threshold was applied at 35 minutes retention time to focus on major peaks. •Colored circles indicate peaks with a fold change (FC) greater than 1.5, with each color representing a corresponding experimental group. *Colored asterisks indicate peaks with FC > 1.5 and p-value < 0.05, with each color representing a corresponding experimental group. The colored peaks in the lower part of the chromatogram represent the injected standard of alkaloids and flavonoids, with purple indicating compounds previously identified in *Erythrina* genus. Each group consisted of *n*=5 biological replicates for leaves and roots, except for control group roots 2 days (*n*=4) (for details see Table S1).


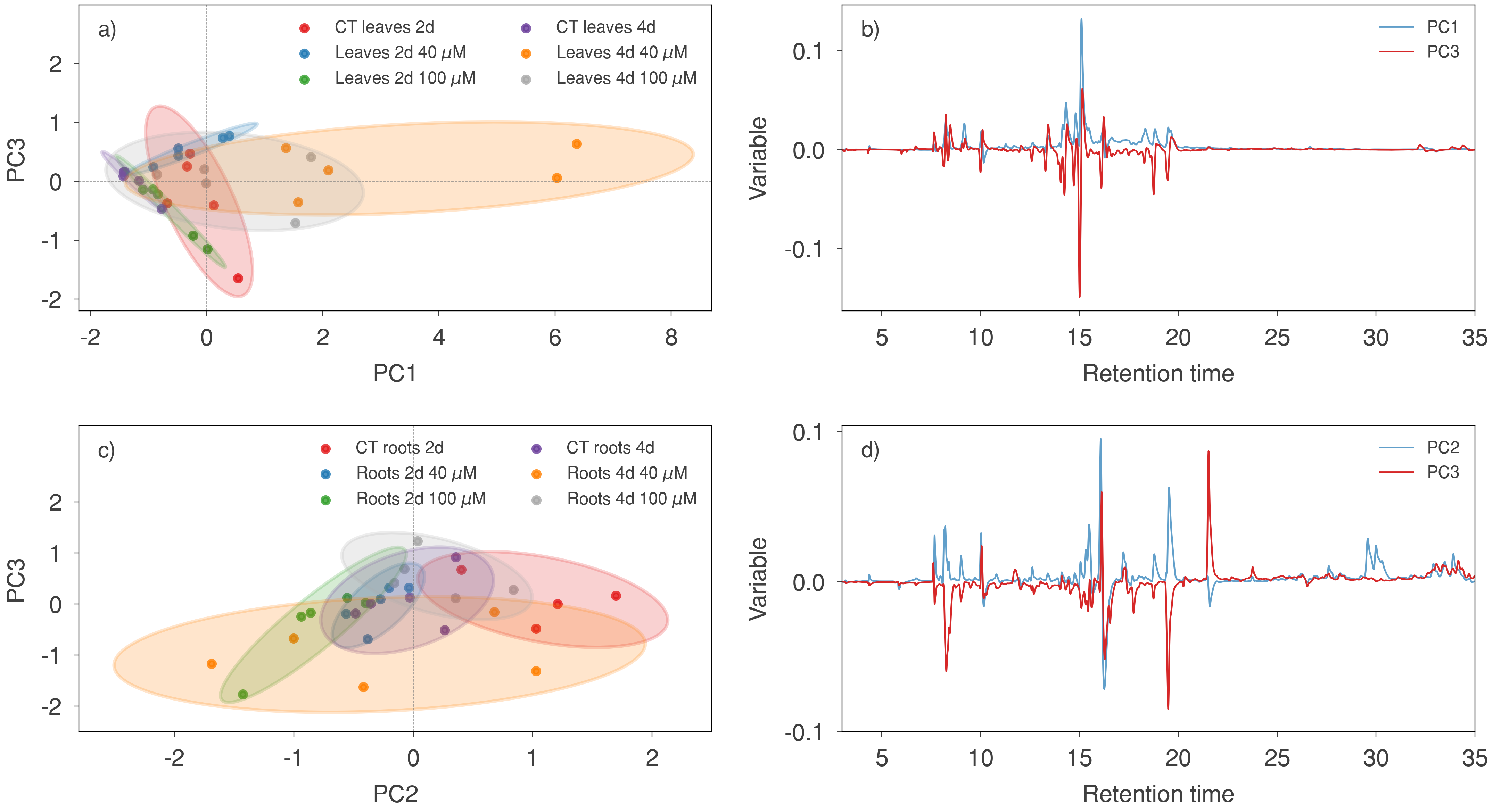


**Figure S14. Plots of scores (left) and loads (right) of ANOVA-PCA results for methyl jasmonate (MeJA) treatment.** MeJA treatment analysis was performed for leaves (a and b) and roots (c and d), for 2 and 4 days and concentration of 40 µM and 100 µM. The first and second principal components are highlighted with green and orange, respectively. The principal components are highlighted with different colors. Ellipse regions in the score graphs represent the region of confidence by class (95%). A threshold was applied at 35 minutes retention time to focus on the main peaks.


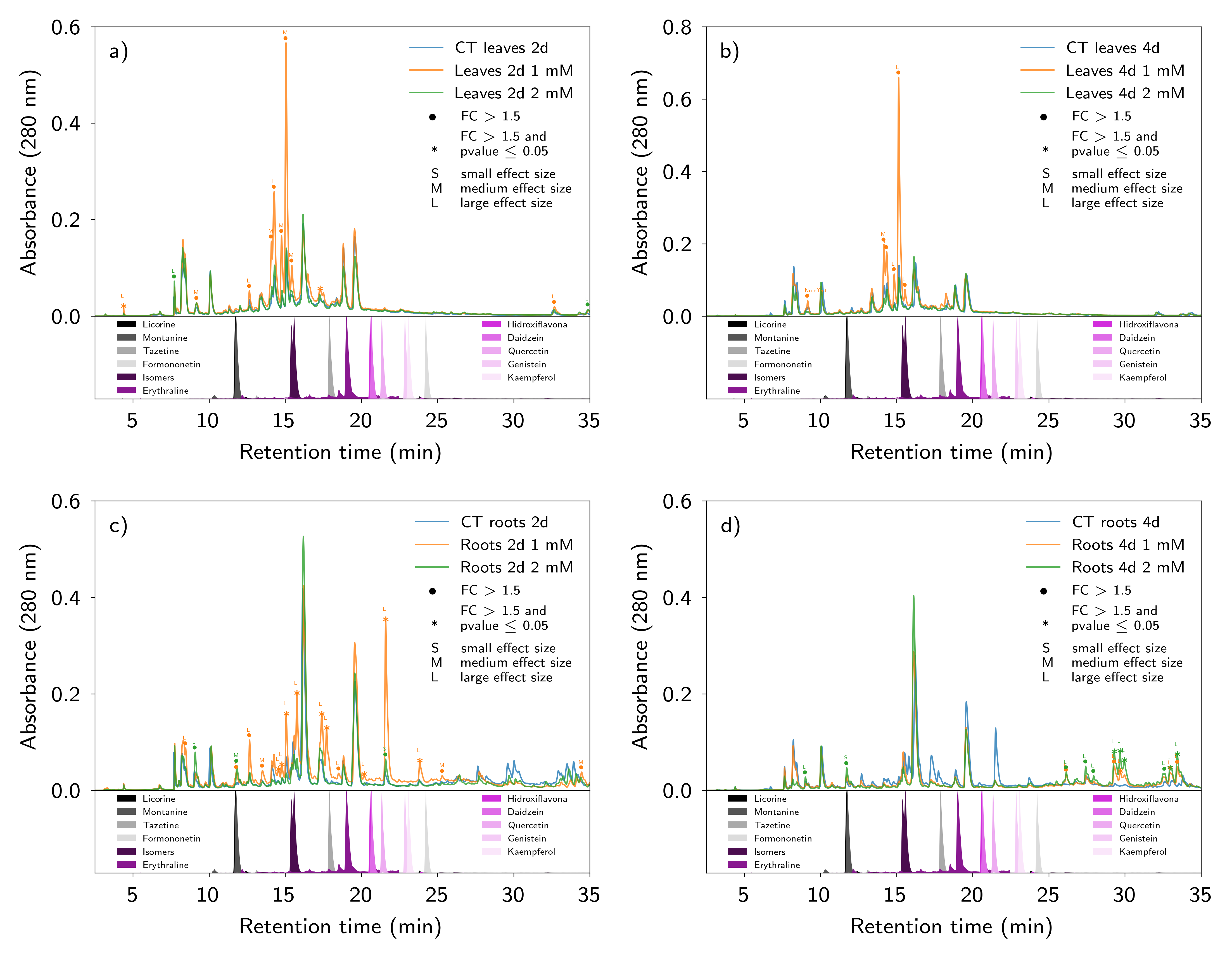


**Figure S15. HPLC fingerprints for salicylic acid (SA) treatment.** Orange and green chromatograms represent the chemical profiles of samples treated with 1 mM and 2 mM of SA, respectively. Blue chromatograms represent controls. (a) and (b) show the chromatograms of leaves at 2 and 4 days, respectively, while (c) and (d) represent the chromatograms of roots at 2 and 4 days after application of SA, respectively. Chromatographic data from replicates were averaged to obtain a single value for each time point, and a threshold was applied at 35 minutes retention time to focus on major peaks. •Colored circles indicate peaks with a fold change (FC) greater than 1.5, with each color representing a corresponding experimental group. *Colored asterisks indicate peaks with FC > 1.5 and p-value < 0.05, with each color representing a corresponding experimental group. The colored peaks in the lower part of the chromatogram represent the injected standard of alkaloids and flavonoids, with purple indicating compounds previously identified in *Erythrina* genus. Each group consisted of *n*=5 biological replicates for leaves and *n*=3 to 5 for roots (for details see Table S1).


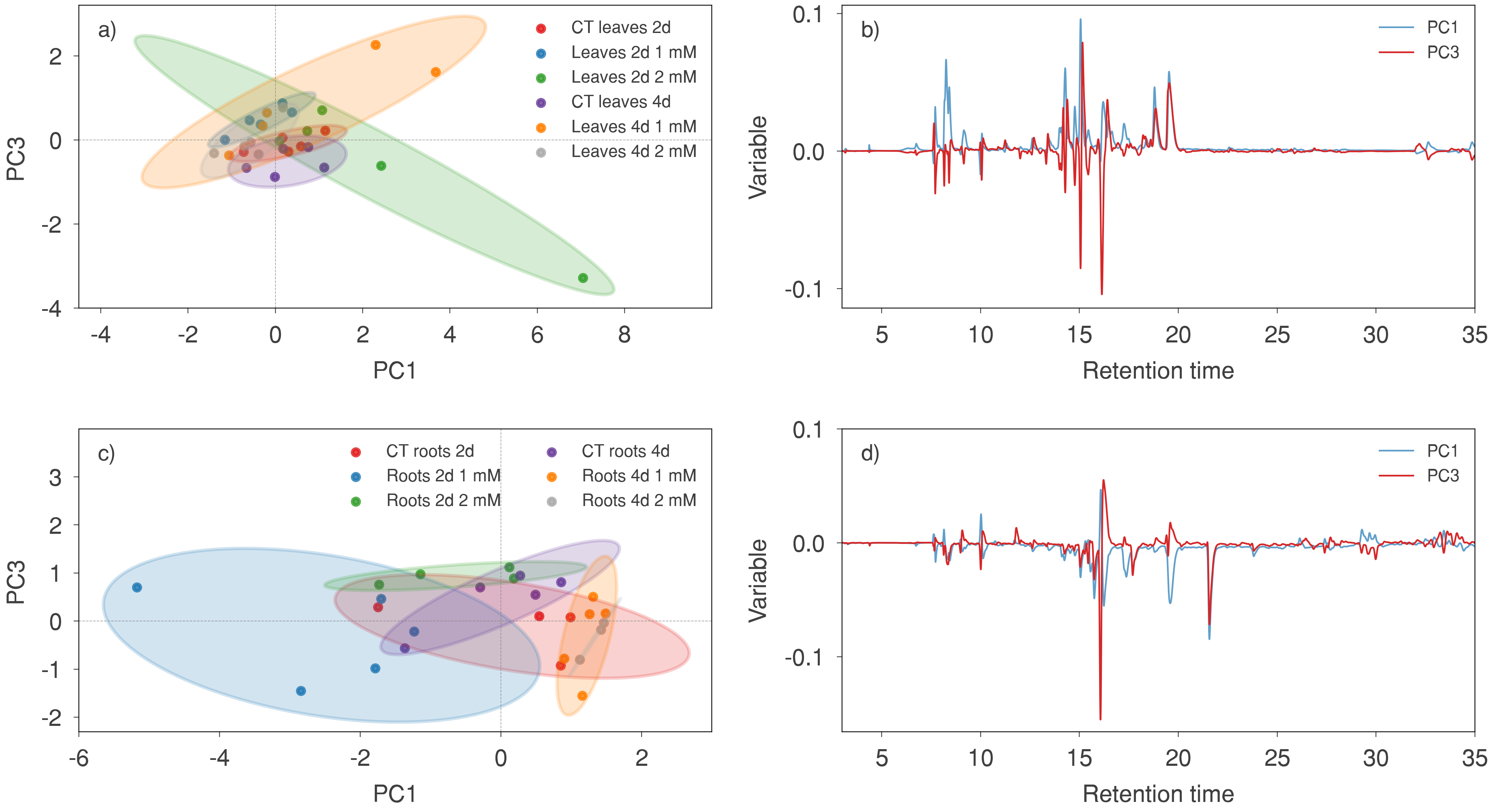


**Figure S16. Plots of scores (left) and loads (right) of ANOVA -PCA results for salicylic acid (SA) treatment.** SA treatment analysis was performed for leaves (a and b) and roots (c and d), for 2 and 4 days and concentration of 1 and 2 mM. The principal components are highlighted with different colors. Ellipse regions in the score graphs represent the region of confidence by class (95%). A threshold was applied at 35 minutes retention time to focus on the main peaks.


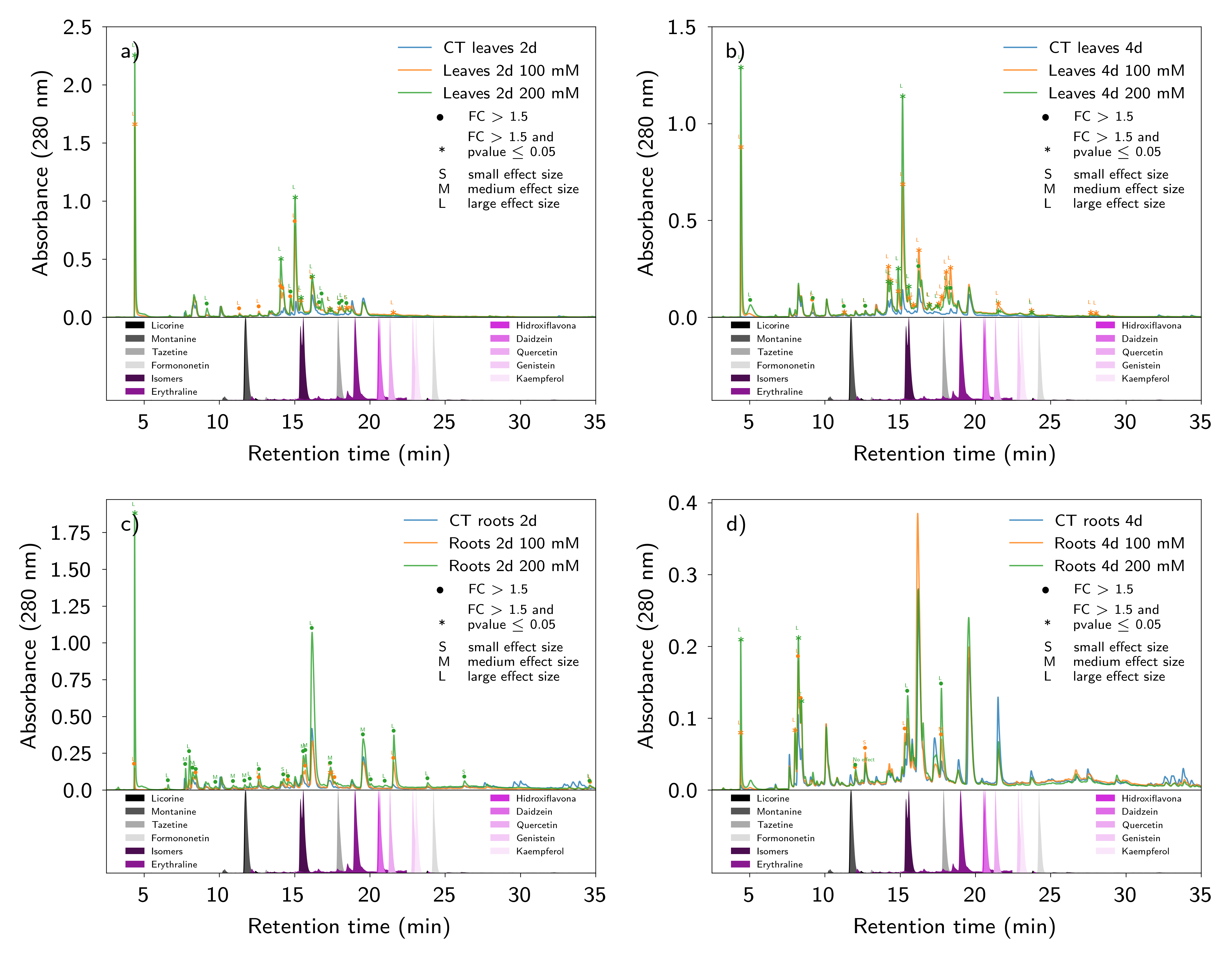


**Figure S17. HPLC fingerprints for nitric oxide (NO) treatment supplied as sodium nitroprusside (SNP).** Orange and green chromatograms represent the chemical profiles of samples treated with 100 mM and 200 mM of SNP, respectively. Blue chromatograms represent controls. (a) and (b) show the chromatograms of leaves at 2 and 4 days, respectively, while (c) and (d) represent the chromatograms of roots at 2 and 4 days after application of SNP, respectively. Chromatographic data from replicates were averaged to obtain a single value for each time point, and a threshold was applied at 35 minutes retention time to focus on major peaks. •Colored circles indicate peaks with a fold change (FC) greater than 1.5, with each color representing a corresponding experimental group. *Colored asterisks indicate peaks with FC > 1.5 and p-value < 0.05, with each color representing a corresponding experimental group. The colored peaks in the lower part of the chromatogram represent the injected standard of alkaloids and flavonoids, with purple indicating compounds previously identified in *Erythrina* genus. Each group consisted of *n*=5 biological replicates for leaves and *n*=4 or 5 for roots (for details see Table S1).


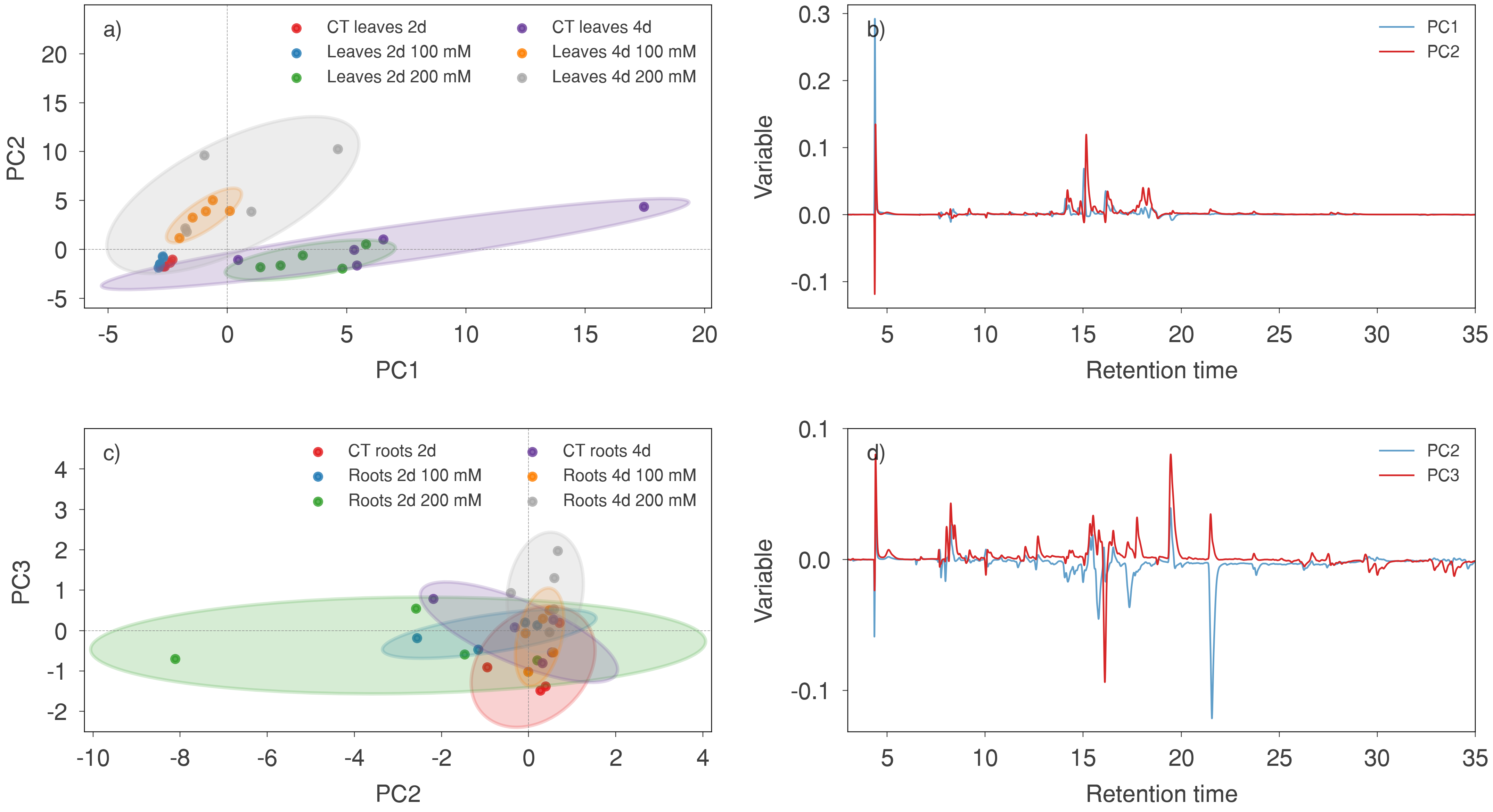


**Figure S18. Plots of scores (left) and loads (right) of ANOVA-PCA results for nitric oxide (NO) treatment supplied as sodium nitroprusside (SNP) treatment.** SNP treatment analysis was performed for leaves (a and b) and roots (c and d), for 2 and 4 days and concentration of 100 and 200 mM. The principal components are highlighted with different colors. Ellipse regions in the score graphs represent the region of confidence by class (95%). A threshold was applied at 35 minutes retention time to focus on the main peaks.


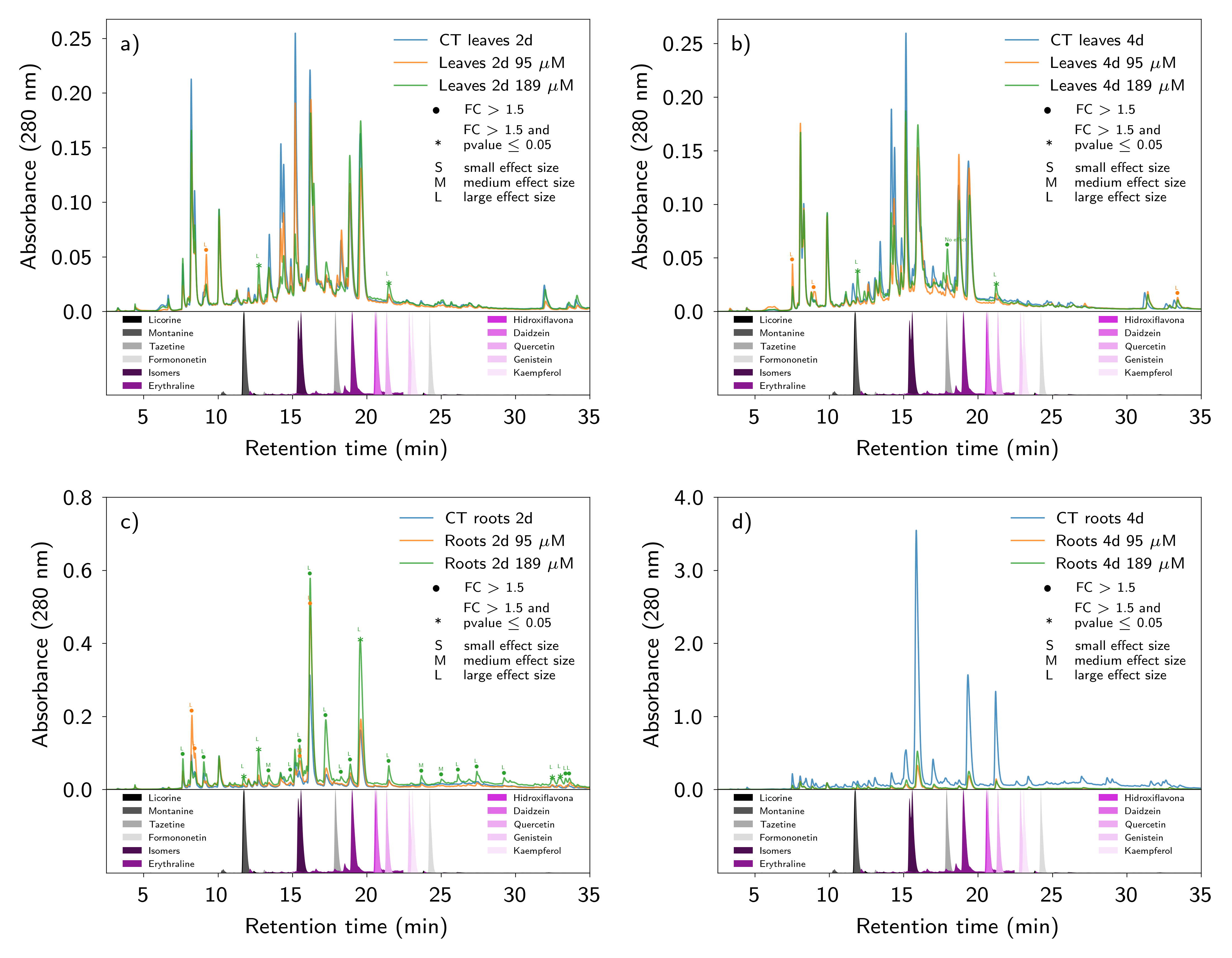


**Figure S19. HPLC fingerprints for abscisic acid (ABA) treatment.** Orange and green chromatograms represent the chemical profiles of the samples treated with 95 µM and 189 µM of ABA, respectively. Blue chromatograms represent controls. (a) and (b) show the chromatograms of leaves at 2 and 4 days, respectively, while (c) and (d) represent the chromatograms of roots at 2 and 4 days after application of ABA, respectively. Chromatographic data from replicates were averaged to obtain a single value for each time point, and a threshold was applied at 35 minutes retention time to focus on major peaks. •Colored circles indicate peaks with a fold change (FC) greater than 1.5, with each color representing a corresponding experimental group. *Colored asterisks indicate peaks with FC > 1.5 and p-value < 0.05, with each color representing a corresponding experimental group. The colored peaks in the lower part of the chromatogram represent the injected standard of alkaloids and flavonoids, with purple indicating compounds previously identified in *Erythrina* genus. Each group consisted of *n*=5 biological replicates for leaves and *n*=3 or 5 for roots (for details see Table S1).


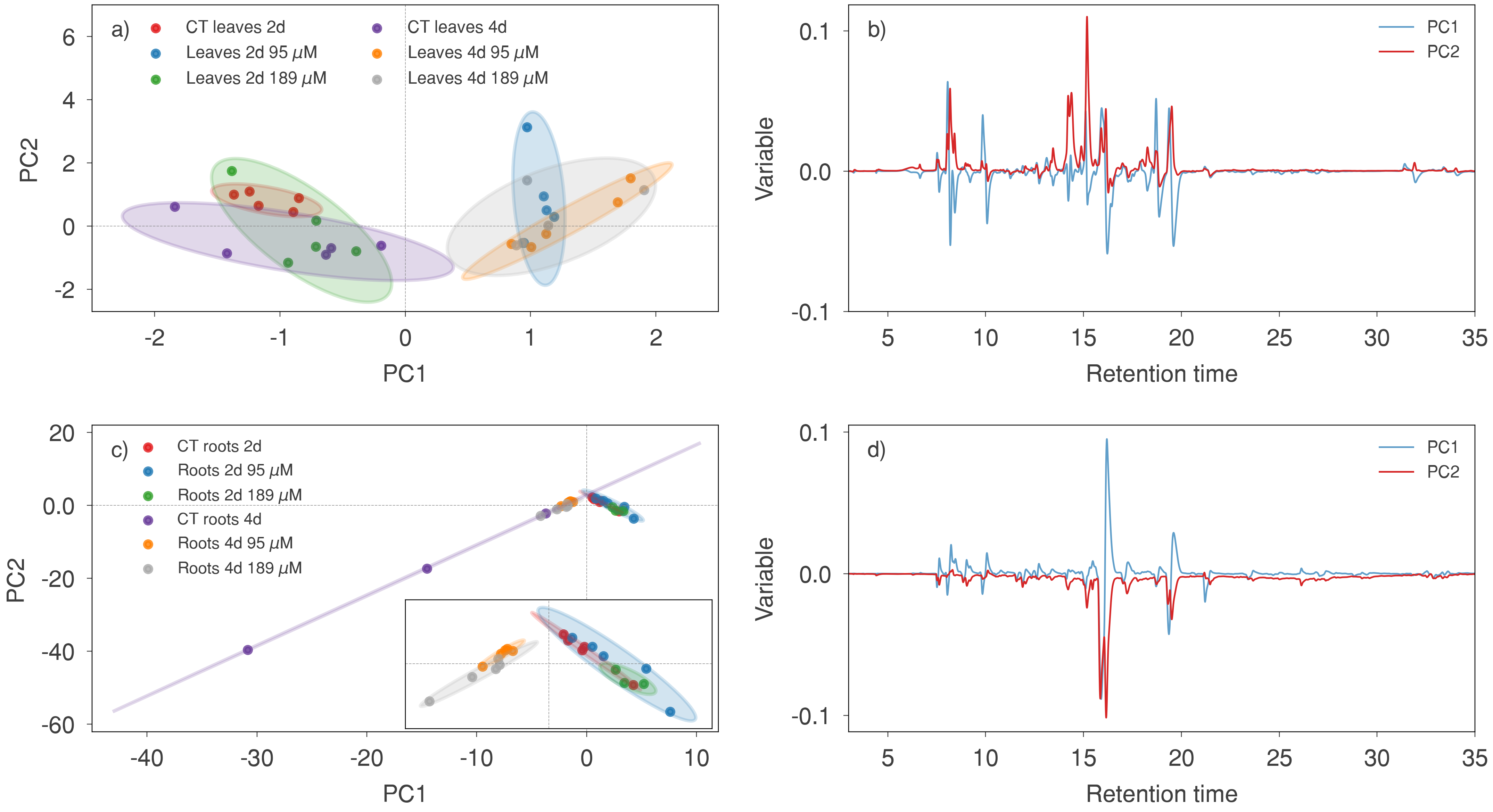


**Figure S20. Plots of scores (left) and loads (right) of ANOVA-PCA results for abscisic acid (ABA) treatment.** ABA treatment analysis was performed for leaves (a and b) and roots (c and d), for 2 and 4 days and concentration of 95 µM and 189 µM. The principal components are highlighted with different colors. Ellipse regions in the score graphs represent the region of confidence by class (95%). A threshold was applied at 35 minutes retention time to focus on the main peaks.


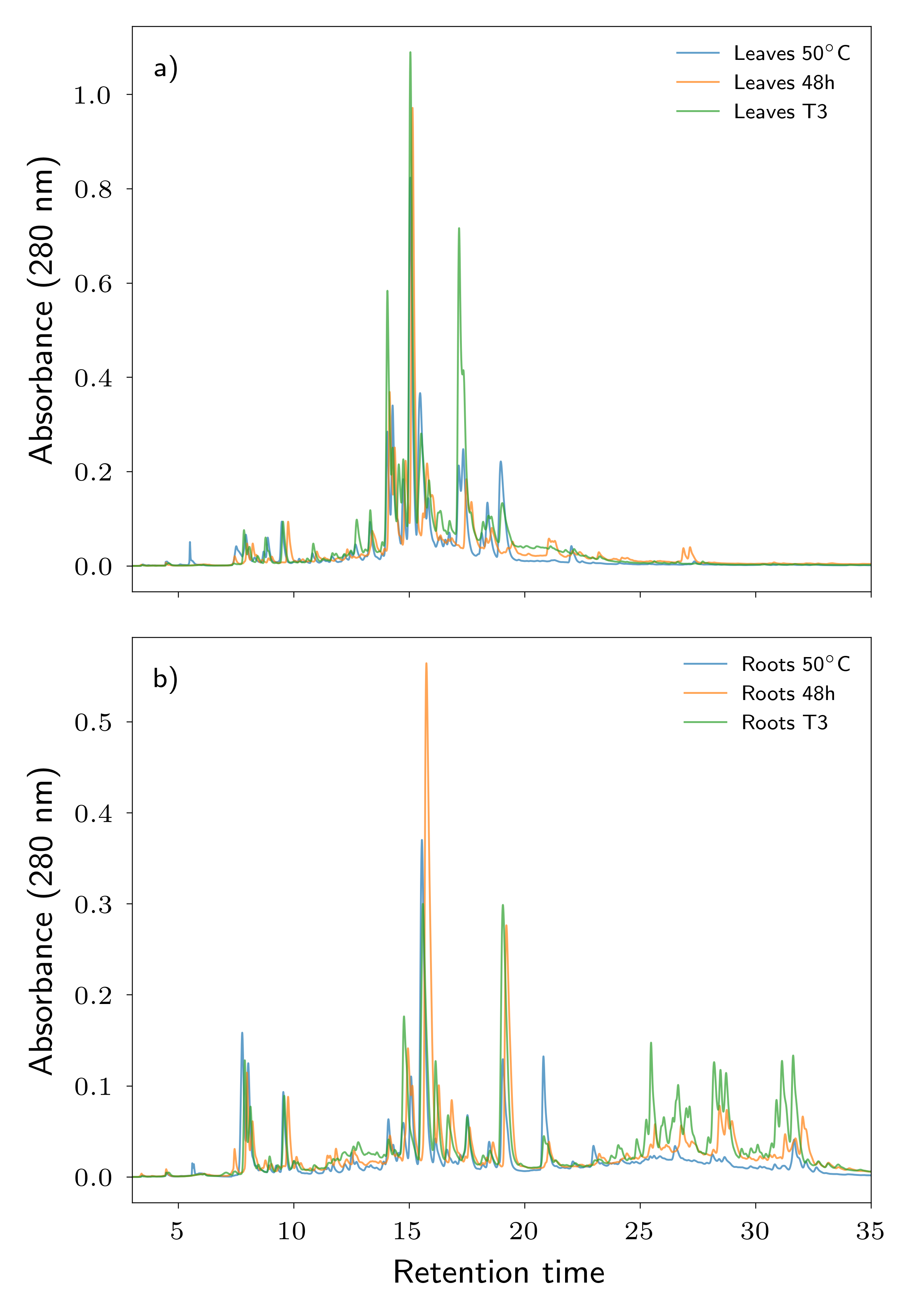


**Figure S21. Overlay graphs of water restriction after 3 weeks (T3), temperature (50°C), and UV light (48h) chromatograms.** (a) and (b) show the chromatograms of leaves and roots respectively. Data from five replicates were averaged to obtain a single value for each time point, and a threshold was applied at 35 minutes retention time to focus on major peaks.


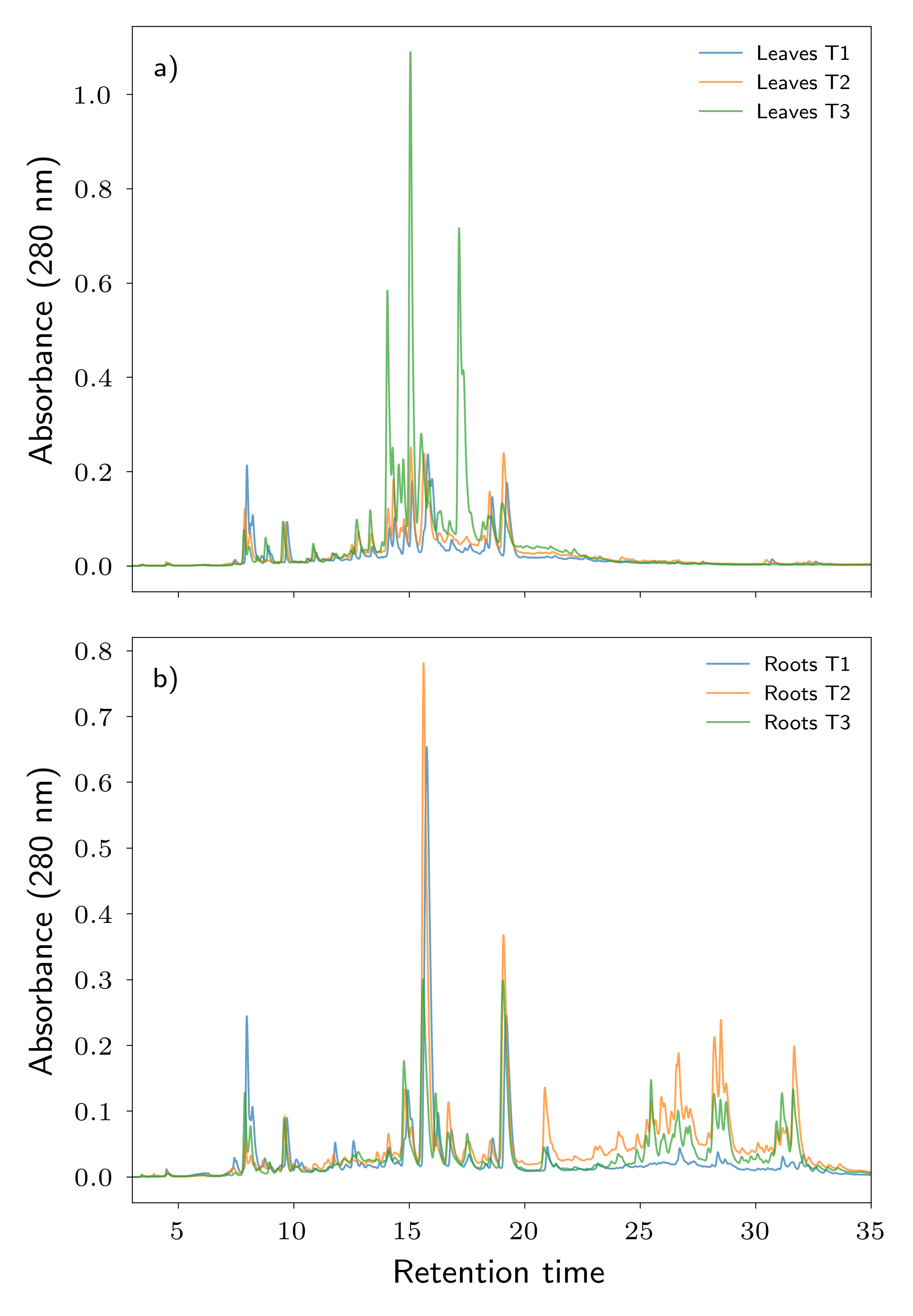


**Figure S22. Overlay graphs of water limitation after one, two and three weeks.** (a) and (b) show the chromatograms of leaves and roots respectively. Data from five replicates were averaged to obtain a single value for each time point, and a threshold was applied at 35 minutes retention time to focus on major peaks.


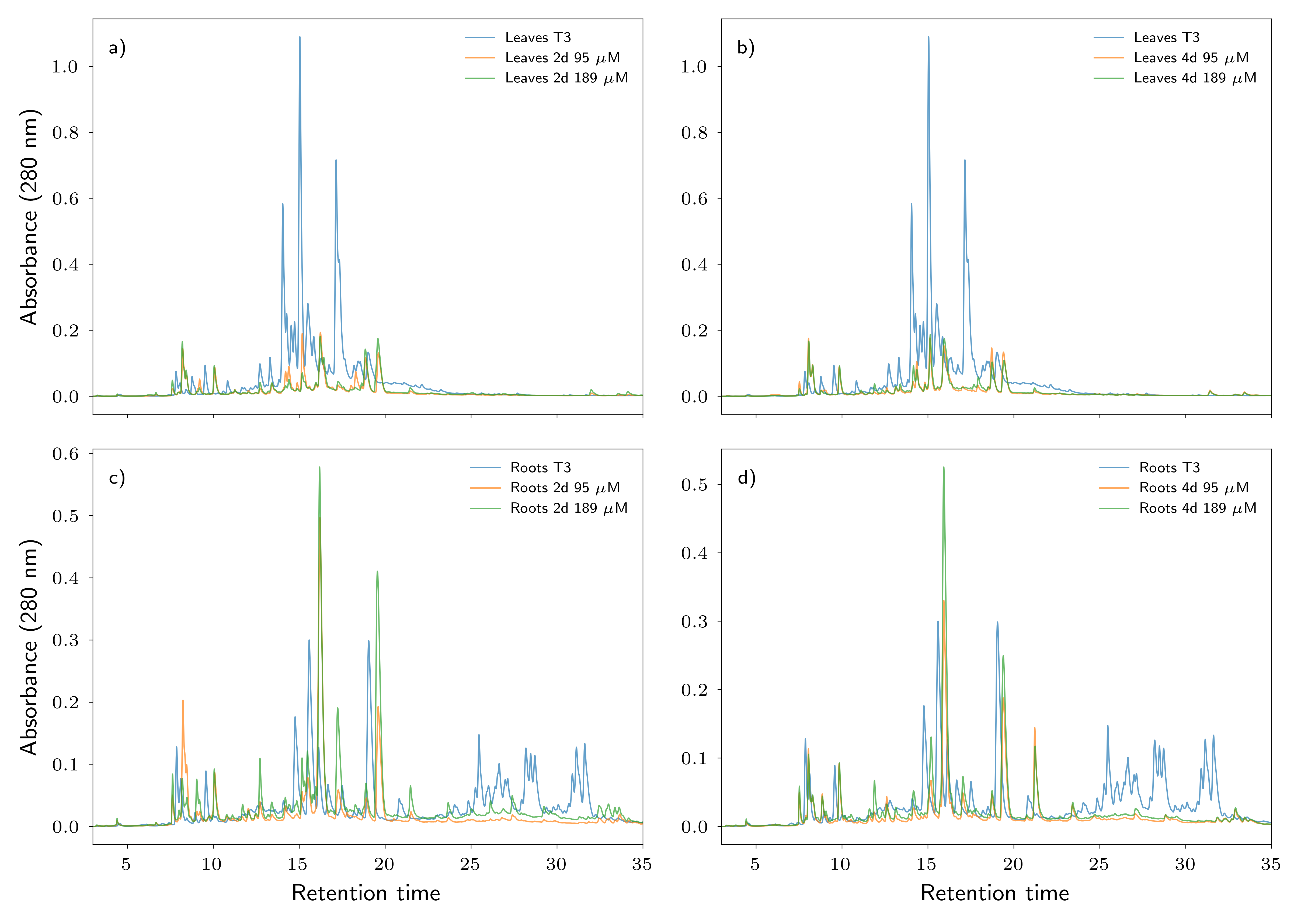


**Figure S23. Overlay graphs of water limitation after three weeks (T3) and ABA 95 μM and 189 μM.** (a) and (b) show the chromatograms of leaves, while (c) and (d) show those of roots. Data from five replicates were averaged to obtain a single value for each time point, and a threshold was applied at 35 minutes retention time to focus on major peaks.


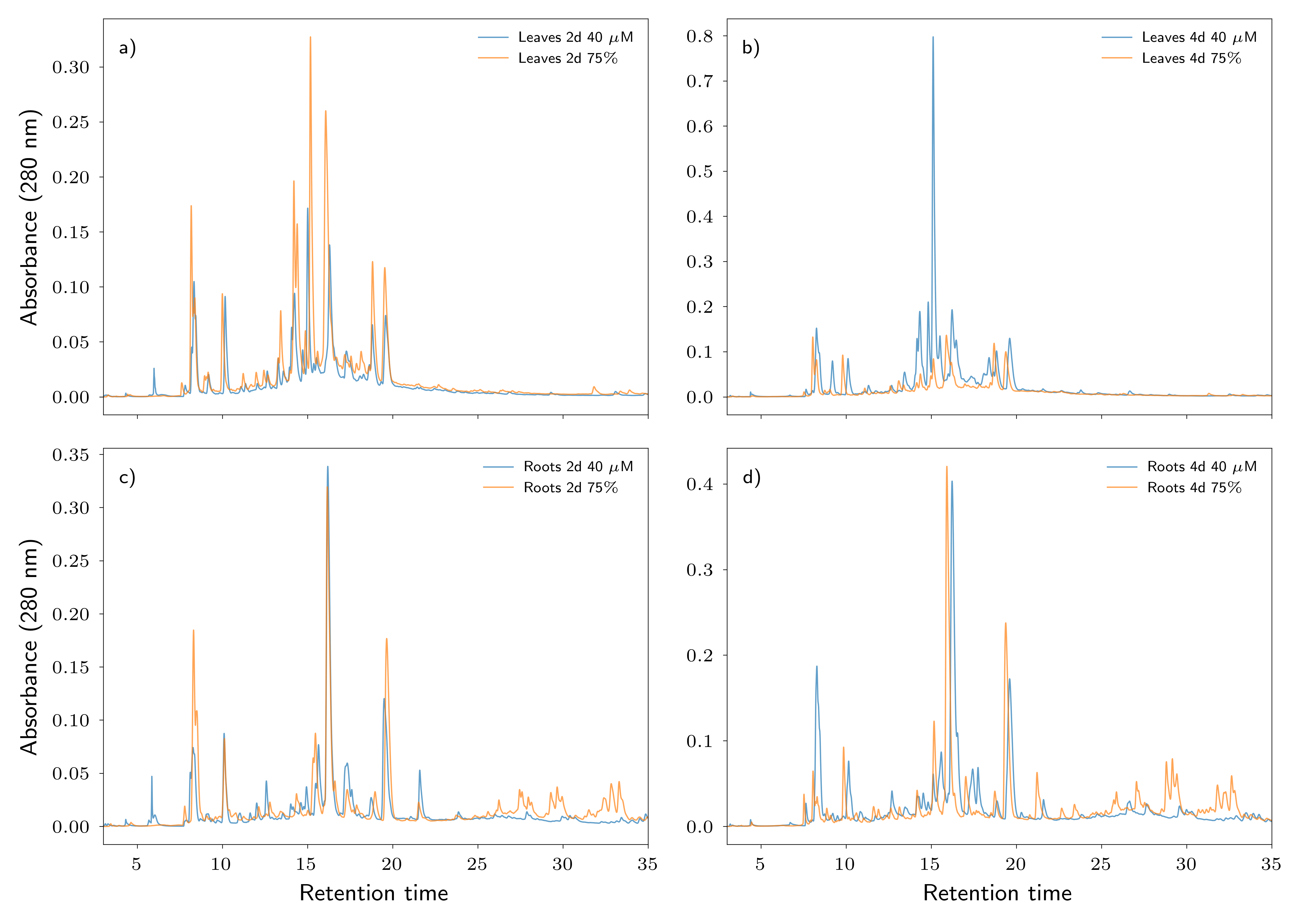


**Figure S24. Overlay graphs of MeJA (40 μM) and mechanical damage (75% of leaf area removal).** (a) and (b) show the chromatograms of leaves and (c) and (d) show those of roots. Data from five replicates were averaged to obtain a single value for each time point, and a threshold was applied at 35 minutes retention time to focus on major peaks.


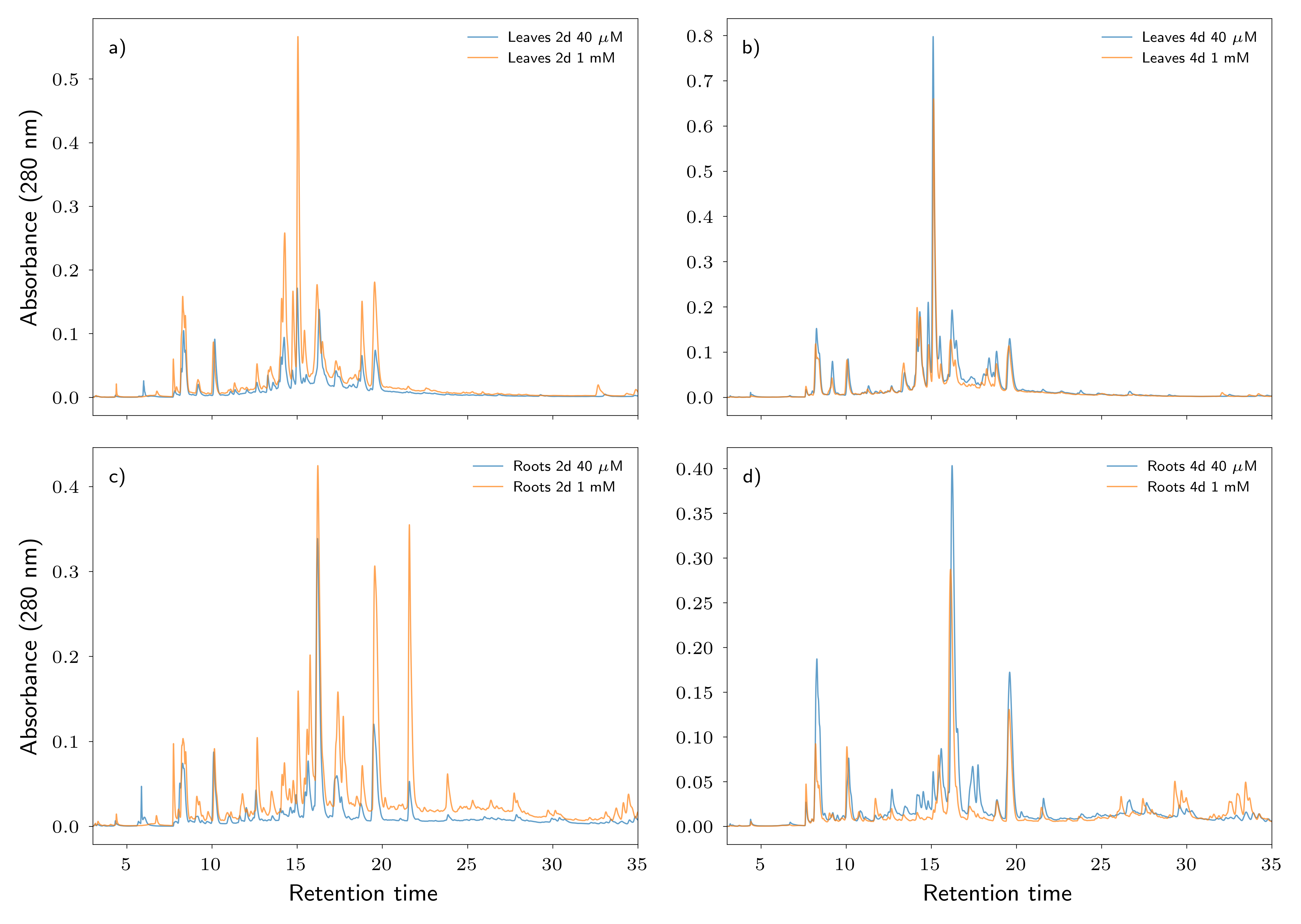


**Figure S25. Overlay graphs of MeJA (40 μM) and Salicylic acid (1 mM).** (a) and (b) show the chromatograms of leaves and (c) and (d) show those of roots. Data from five replicates were averaged to obtain a single value for each time point, and a threshold was applied at 35 minutes retention time to focus on major peaks.

**
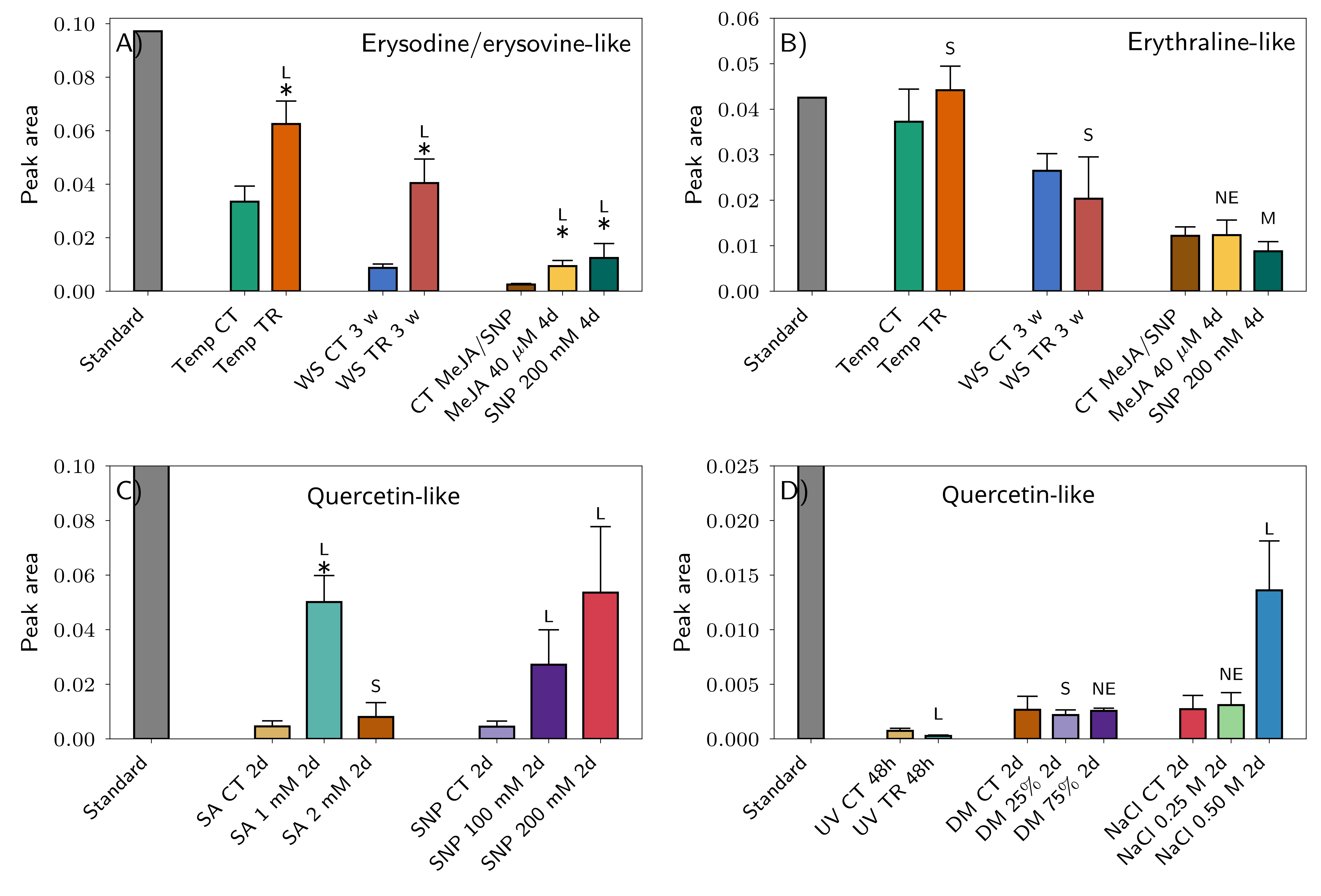
**

**Figure S26. Bar plots showing the chromatographic peak areas of compounds classified as (A) Erysodine/Erysovine-like, (B) Erythraline-like, and (C, D) Quercetin-like under different experimental conditions.** Values are expressed as mean ± standard error. Letters denote effect sizes (S = small, M = medium, L = large, NE = no effect). Asterisks indicate statistically significant differences between treated groups and their respective controls (p < 0.05). Colored bars indicate peak area in the experimental groups, and gray bars indicate peak area of ​​the injected standard.


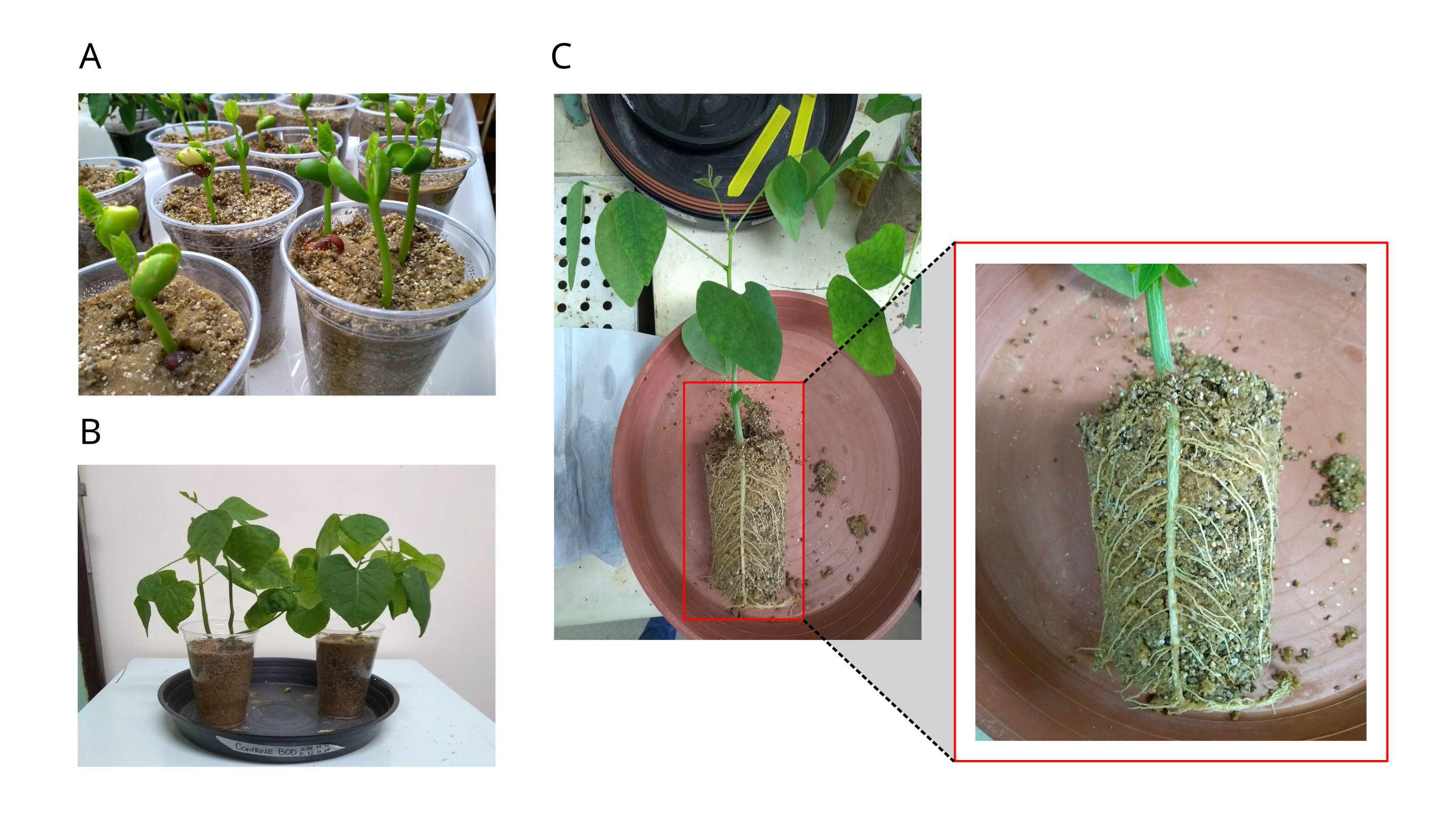


**Scheme S1. Developmental series of seed germination and growth of *Erythrina velutina*.** Seedlings after germination (A); Control seedlings during experiment (B); Seedling roots at the end of the experiment (C).

**Table S1.** Experimental design of treatments for *Erythrina velutina* leaves and roots.

| **Elicitor** | **Plant organ** | **Harvest days** | **Factor variables** | **Biological replicates x**  **technical** | **Total analysis/factor** | **Total analysis** |
| --- | --- | --- | --- | --- | --- | --- |
| **Methyl jasmonate**  **(MeJA)** | Leaves | 2 days | 40 uM | 5 x 2 | 20 | **80** |
|  |  | 4 days |  | 5 x 2 |  |  |
|  |  | 2 days | 100 uM | 5 x 2 | 20 |  |
|  |  | 4 days |  | 5 x 2 |  |  |
|  | Roots | 2 days | 40 uM | 5 x 2 | 20 |  |
|  |  | 4 days |  | 5 x 2 |  |  |
|  |  | 2 days | 100 uM | 5 x 2 | 20 |  |
|  |  | 4 days |  | 5 x 2 |  |  |
| **Salicylic acid (SA)** | Leaves | 2 days | 1 mM | 5 x 2 | 20 | **74** |
|  |  | 4 days |  | 5 x 2 |  |  |
|  |  | 2 days | 2 mM | 5 x 2 | 20 |  |
|  |  | 4 days |  | 5 x 2 |  |  |
|  | Roots | 2 days | 1 mM | 5 x 2 | 20 |  |
|  |  | 4 days |  | 5 x 2 |  |  |
|  |  | 2 days | 2 mM | 4 x 2 | 14 |  |
|  |  | 4 days |  | 3 x 2 |  |  |
| **Sodium nitroprusside**  **(SNP)** | Leaves | 2 days | 100 mM | 5 x 2 | 20 | **76** |
|  |  | 4 days |  | 5 x 2 |  |  |
|  |  | 2 days | 200 mM | 5 x 2 | 20 |  |
|  |  | 4 days |  | 5 x 2 |  |  |
|  | Roots | 2 days | 100 mM | 4 x 2 | 18 |  |
|  |  | 4 days |  | 5 x 2 |  |  |
|  |  | 2 days | 200 mM | 4 x 2 | 18 |  |
|  |  | 4 days |  | 5 x 2 |  |  |
|  |  | 4 days |  |  |  |  |
| **Control**  **MeJA + SA +**  **SNP** | Leaves | 2 days | - | 5 x 2 | 20 | **38** |
|  |  | 4 days | - | 5 x 2 |  |  |
|  | Roots | 2 days | - | 4 x 2 | 18 |  |
|  |  | 4 days | - | 5 x 2 |  |  |
| **Mechanical damage (MD)** | Leaves | 2 days | 25% | 5 x 2 | 20 | **76** |
|  |  | 4 days |  | 5 x 2 |  |  |
|  |  | 2 days | 75% | 5 x 2 | 20 |  |
|  |  | 4 days |  | 5 x 2 |  |  |
|  | Roots | 2 days | 25% | 5 x 2 | 16 |  |
|  |  | 4 days |  | 3 x 2 |  |  |
|  |  | 2 days | 75% | 5 x 2 | 20 |  |
|  |  | 4 days |  | 5 x 2 |  |  |
| **Saline stress with sodium chloride**  **(NaCl)** | Leaves | 2 days | 0,25 M | 2 x 2 | 14 | **66** |
|  |  | 4 days |  | 5 x 2 |  |  |
|  |  | 2 days | 0,50 M | 5 x 2 | 20 |  |
|  |  | 4 days |  | 5 x 2 |  |  |
|  | Roots | 2 days | 0,25 M | 3 x 2 | 16 |  |
|  |  | 4 days |  | 5 x 2 |  |  |
|  |  | 2 days | 0,50 M | 5 x 2 | 16 |  |
|  |  | 4 days |  | 3 x 2 |  |  |
| **Abscisic acid (ABA)** | Leaves | 2 days | 95 uM | 5 x 2 | 20 | **76** |
|  |  | 4 days |  | 5 x 2 |  |  |
|  |  | 2 days | 189 uM | 5 x 2 | 20 |  |
|  |  | 4 days |  | 5 x 2 |  |  |
|  | Roots | 2 days | 95 uM | 5 x 2 | 20 |  |
|  |  | 4 days |  | 5 x 2 |  |  |
|  |  | 2 days | 189 uM | 3 x 2 | 16 |  |
|  |  | 4 days |  | 5 x 2 |  |  |
| **Control MD + NaCl + ABA** | Leaves | 2 days | - | 5 x 2 | 20 | **36** |
|  |  | 4 days | - | 5 x 2 |  |  |
|  | Roots | 2 days | - | 5 x 2 | 16 |  |
|  |  | 4 days | - | 3 x 2 |  |  |
| **UV light** | Leaves | 24 h | | 5 x 2 | 20 | **36** |
|  |  | 48 h | | 5 x 2 |  |  |
|  | Roots | 24 h | | 4 x 2 | 16 |  |
|  |  | 48 h | | 4 x 2 |  |  |
| **Control UV** | Leaves | 24 h | | 5 x 2 | 20 | **40** |
|  |  | 48 h | | 5 x 2 |  |  |
|  | Roots | 24 h | | 5 x 2 | 20 |  |
|  |  | 48 h | | 5 x 2 |  |  |
| **Temperature** | Leaves | 6 h | 50 ºC | 5 x 2 | 20 | **40** |
|  | Roots | 6 h | 50 ºC | 5 x 2 |  |  |
| **Control Temperature** | Leaves | 6 h | 24 ºC | 5 x 2 | 20 |  |
|  | Roots | 6 h | 24 ºC | 5 x 2 |  |  |
| **Drought stress (DS)** | Leaves | T0 | | 5 X 2 | 40 | **76** |
|  |  | T1 | | 5 X 2 |  |  |
|  |  | T2 | | 5 X 2 |  |  |
|  |  | T3 | | 5 X 2 |  |  |
|  | Roots | T0 | | 5 X 2 | 36 |  |
|  |  | T1 | | 5 X 2 |  |  |
|  |  | T2 | | 3 X 2 |  |  |
|  |  | T3 | | 5 X 2 |  |  |
| **Control DS** | Leaves | C1 | | 5 X 2 | 30 | **56** |
|  |  | C2 | | 5 X 2 |  |  |
|  |  | C3 | | 5 X 2 |  |  |
|  | Roots | C1 | | 4 x 2 | 26 |  |
|  |  | C2 | | 4 x 2 |  |  |
|  |  | C3 | | 5 x 2 |  |  |
| **Total analysis** | | | | | | 770 |
| **Total replicates** | | | | | | 385 |

**Table S2**. Mobile phase gradient used for experiments in HPLC-DAD

| **Time** | **Solution A** | **Solution B** |
| --- | --- | --- |
| 00 min → | 100 % | 0 % |
| 15 min → | 65 % | 35 % |
| 23 min → | 35 % | 65 % |
| 40 min → | 0 % | 100 % |
| 48 min → | 0 % | 100 % |
| 50 min → | 100 % | 0 % |
| 60 min → | 100 % | 0 % |

**Table S3**. Patterns of alkaloids and flavonoids injected into HPLC-DAD. RT: retention time in minutes

# Metabolite Group Class RT

| Lycorine | Non-*Erythrine* alkaloid | Alkaloid | 11.713 |
| --- | --- | --- | --- |
| Montanine | Non-*Erythrine* alkaloid | Alkaloid | 11.753 |
| Isomer 1 | *Erythrine* alkaloid | Alkaloid | 15.38 |
| Isomer 2 | *Erythrine* alkaloid | Alkaloid | 15.563 |
| Tazetine | Non-*Erythrine* alkaloid | Alkaloid | 17.877 |
| Erythraline | *Erythrine* alkaloid | Alkaloid | 19 |
| 7,4 Hydroxyflavone | Flavone | Flavonoid | 20.546 |
| Daidzein | Isoflavone | Flavonoid | 20.679 |
| Quercetin | Flavonol | Flavonoid | 21.32 |
| Genistein | Isoflavone | Flavonoid | 22.839 |
| Kaempferol | Flavonol | Flavonoid | 23.059 |
| Formononetin | Isoflavone | Flavonoid | 24.213 |

**Table S4**. Treatment table and evaluation of alignment/non-alignment

| **Treatment** | **Situation** |
| --- | --- |
| TEMPERATURE | Not aligned |
| UV | Not aligned |
| DROUGHT STRESS | Only CT1+T1 and T0+T2 aligned |
| SALINE STRESS | Only leaves 2d (CT+TR) and roots 2d (CT+TR) aligned |
| MECHANICAL DAMAGE | Only roots 2d (CT+TR) aligned |
| METHYL JASMONATE | Only leaves 2d (CT+TR) aligned |
| SODIUM NITROPRUSSIDE | Only leaves 4d (CT+TR) aligned |
| SALICYLIC ACID | Not aligned |
| ABSCISIC ACID | Not aligned |
